# Genetically Engineered Light‐Responsive In Situ Hydrogels for Immunomodulation and Multimodal Therapy in Metastatic Triple‐Negative Breast Cancer

**DOI:** 10.1002/advs.202512355

**Published:** 2025-10-29

**Authors:** Xinchen Shen, Jiajia Zhang, Junhan Ou, Ziheng Bai, Tongyan Liu, Kaiyue Zhang, Kaixiang Zhu, James Q. Wang, Chaochen Wang, Qianting Zhang, Linrong Lu, Wenwen Huang

**Affiliations:** ^1^ The Zhejiang University‐University of Edinburgh Institute Zhejiang University School of Medicine Zhejiang University Hangzhou 310058 China; ^2^ Deanery of Biomedical Sciences Edinburgh Medical School College of Medicine and Veterinary Medicine The University of Edinburgh Edinburgh EH89AG UK; ^3^ Department of Cardiology of the Second Affiliated Hospital Zhejiang University School of Medicine Zhejiang University Hangzhou 310058 China; ^4^ Department of Orthopedics of the Second Affiliated Hospital Zhejiang University School of Medicine Zhejiang University Hangzhou 310058 China; ^5^ Dr. Li Dak Sum & Yip Yio Chin Center for Stem Cells and Regenerative Medicine Zhejiang University School of Medicine Zhejiang University Hangzhou 310058 China; ^6^ State Key Laboratory of Biobased Transportation Fuel Technology Zhejiang University Hangzhou 310027 China; ^7^ Biomedical and Health Translational Research Centre of Zhejiang Province Zhejiang University Hangzhou 310003 China

**Keywords:** chimeric proteins, in situ hydrogels, on‐demand drug release, stimuli‐responsive hydrogels, triple‐negative breast cancer

## Abstract

Triple‐negative breast cancer (TNBC) is a highly aggressive subtype of breast cancer, known for its early onset, strong metastatic tendencies, and poor prognosis. Conventional treatments, including chemotherapy and immunotherapy, often face challenges such as limited efficacy, adverse side effects, and high recurrence rates. To address these limitations, a family of tri‐block chimeric proteins, cysteine‐tagged silk‐elastin‐like proteins (cSELPs), is designed to form responsive in situ hydrogels for treating late‐stage and metastatic TNBC. These cSELPs are de novo designed to integrate multiple functional protein motifs, including a photothermal agent binding motif, silk‐inspired crosslinking motifs, and elastin‐like thermo‐responsive motifs. This unique sequence design enables the cSELP hydrogels to exhibit in situ gelation, photothermal responsive release of chemotherapeutic agent doxorubicin and immune checkpoint inhibitor anti‐PD‐L1, and antibacterial properties, leading to effective tumor microenvironment remodeling. By promoting immunogenic cell death and stimulating immune activation, this approach converts immunosuppressive “cold” tumors into immunologically active “hot” tumors. The cSELP hydrogel system demonstrates potent therapeutic efficacy against both primary and metastatic TNBC while maintaining excellent biocompatibility and long‐term safety. This protein material platform offers an innovative strategy to reshape the tumor microenvironment and combine multimodal treatments into a single biocompatible system, highlighting its potential for clinical translation.

## Graphical abstract

Cysteine‐tagged silk‐elastin‐like proteins, a family of tri‐block chimeric proteins that consist of a photothermal agent binding motif, silk motif, and elastin motif is developed to form a responsive in situ hydrogel for late‐stage and metastatic triple‐negative breast cancer. These hydrogels support the light‐controlled, sustained release of doxorubicin and anti‐PD‐L1, enhancing therapeutic outcomes through tumor microenvironment remodeling and showing better suppression efficacy for larger tumor models and distal tumors.

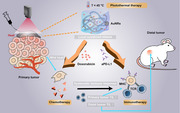

## 1 Introduction

Cancer remains a significant public health challenge in the 21st century. According to GLOBOCAN estimates, almost 10 million people died from malignant tumors in 2022.^[^ ^1^ ^]^ Notably, female breast cancer incidences have been slowly increasing every year since the mid‐2000s, making it the second most common cancer worldwide.^[^ ^2^ ^]^ Triple‐negative breast cancer (TNBC), which accounts for 15–20% of incident breast cancers,^[^ ^3^ ^]^ is the most aggressive type of all breast cancers. Compared with other invasive breast cancers, TNBC tends to have an earlier age of onset, grows and spreads faster, and generally has a worse prognosis with a higher rate of recurrence.^[^ ^4^ ^]^ As it is negative for estrogen receptor (ER), progesterone receptor (PR), and human epidermal growth factor receptor 2 (HER2), TNBC has very limited treatment options.^[^ ^5^ ^]^ The primary treatment of TNBC patients is chemotherapy due to the high chemosensitivity of TNBC tumors,^[^ ^6^ ^]^ but TNBC is prone to rapid recurrence and drug resistance, referring to the triple negative paradox.^[^ ^7^ ^]^ Immune checkpoint blockade (ICB) is emerging as a promising cancer immunotherapy that aims to reshape the immunosuppressive tumor microenvironment (TME) using immune checkpoint inhibitors. TNBC has a higher expression of the programmed death‐ligand protein (PD‐L1) compared to other breast cancer types,^[^ ^8^ ^]^ thereby monoclonal antibodies anti‐PD‐L1 (aPD‐L1) have been widely used to block overexpressed PD‐L1 on the surface of TNBC cells to enhance T cell activation and cytotoxicity.^[^ ^9^ ^]^ Though TNBC demonstrates better immunotherapy response compared to other breast cancer subtypes, the prognosis of TNBC patients remains unsatisfactory in the clinic, especially since immunotherapy and chemo‐immunotherapy only demonstrate better suppression efficacy for early TNBC.^[^ ^10^ ^]^ Until now, there is still an unmet need to design effective therapy targeting late‐stage and metastatic TNBC.

In recent years, various site‐specific drug delivery systems have emerged as viable alternatives to systemic therapies, offering safer and more effective cancer treatments.^[^ ^11^, ^12^, ^13^ ^]^ Nanomedicines designed with the function of active targeting are the most investigated systems for realizing this pursuit, showing considerable progress in diagnostic and therapeutic applications.^[^ ^14^ ^]^ However, low enhanced permeabilization and retention (EPR) effect,^[^ ^15^ ^]^ lack of understanding of nanomedicine‐bio interactions,^[^ ^16^ ^]^ limited loading capacity,^[^ ^17^ ^]^ modest tumor accumulation due to multiple biological barriers,^[^ ^18^ ^]^ and pharmacokinetic problems^[^ ^19^ ^]^ have limited the effectiveness of nanomedicines. Meta‐analyses indicated that administering drugs in the proximity of the lesion exhibited higher drug concentration at the tumor site and lower systemic toxicity compared to systemic administration (oral, intraperitoneal, or intravenous).^[^ ^20^ ^]^ Moreover, the advancements in real‐time imaging and surgical technology ensure that most parts of a cancer patient can be precisely accessed to implement locoregional drug delivery,^[^ ^21^ ^]^ resulting in significantly increased clinical feasibility of this approach. Therefore, locoregional drug delivery, which bypasses blood circulation, has become a more promising strategy for cancer therapy.^[^ ^22^ ^]^

In situ hydrogels, a class of highly hydrated 3D porous networks that form upon injection, have shown great prospects as locoregional delivery systems for cancer treatment.^[^ ^23^, ^24^ ^]^ Beyond the conventional advantages of hydrogels, in situ hydrogels offer significantly prolonged drug retention at targeted pathological sites and exhibit minimally invasive properties, thus reducing the need for repeated drug administration and enhancing patient tolerance.^[^ ^25^ ^]^ Moreover, multi‐responsive functionalities have been incorporated into hydrogel systems to further optimize pharmacokinetics and therapeutic outcomes.^[^ ^26^ ^]^ To date, several thermo‐responsive polymers capable of sol–gel transition at body temperature have been explored for in situ gelling.^[^ ^27^ ^]^ Although considerable progress has been demonstrated, traditional thermo‐responsive materials, such as poly(N‐isopropylacrylamide), still require optimization due to the complex synthesis processes and limited stimuli‐responsiveness to environmental or biological signals.^[^ ^28^, ^29^ ^]^ Therefore, developing intrinsically biocompatible hydrogels that are relatively easy to synthesize, more versatile in functionalization, and tunable in responsive properties is attractive for creating in situ, responsive hydrogel systems as effective locoregional drug delivery carriers.

With the rapid advancement in synthetic biology and computational modeling,^[^ ^30^, ^31^, ^32^ ^]^ de novo protein sequences now offer expanded opportunities to engineer recombinant biomaterials with precisely tunable properties and functionalities that surpass their natural counterparts.^[^ ^33^, ^34^, ^35^, ^36^, ^37^ ^]^ Herein, inspired by the natural fibrous proteins, we designed cysteine‐tagged silk‐elastin‐like proteins (cSELPs), a family of tri‐block chimeric proteins that consist of silk motif, elastin motif, and photothermal agent binding motif, to develop responsive in situ hydrogels for the multimodal therapy for late‐stage and metastatic TNBC. Specifically, the SELP His‐tag was mutated to include a cysteine residue, enabling site‐specific interaction with gold nanorods (AuNRs). Although this adjustment was simple, it substantially enhanced the material functionality by imparting stable photothermal responsiveness under near‐infrared (NIR) light. This allowed precise, on‐demand drug release from the cSELP hydrogel matrix. Furthermore, the protein coating offered a biocompatible interface that stabilized the AuNRs and ensured their safe application in vivo. The elastin motif was designed with enzymatic crosslinking sites and thermo‐responsiveness to facilitate the localized formation of in situ photothermal hydrogel depots.^[^ ^38^ ^]^ The silk motifs served as dual crosslinking sites for a sustainable release of the loaded chemotherapy drug doxorubicin (DOX) and immune checkpoint inhibitor anti‐PD‐L1. This chimeric protein design enables a synergistic photothermal‐triggered chemo‐immunotherapy (SPTCI), showing better suppression efficacy for larger tumor models (average starting volume of ≈150 mm^3^)^[^ ^39^ ^]^ and distal tumors. Meanwhile, the chimeric protein‐based SPTCI strategy possesses high biosafety, achieving an effective tumor‐suppressing effect with low doses of drugs and a short irradiation time of 3 min. Overall, by activating the systemic anti‐tumor immune response, the current study offers a versatile and biocompatible platform that can successfully modulate primary and distal tumor TMEs by increasing T cell infiltration, turning “cold” tumors into “hot” tumors, thus realizing abscopal effects under locoregional treatment. In this study, we developed a broadly applicable in situ hydrogel platform based on a rationally engineered protein backbone with intrinsic thermo‐responsiveness and biocompatibility. Our approach focused on enhancing the local pharmacological performance of clinically approved agents through controlled release and improved retention. This strategy enabled effective treatment of TNBC and holds promise for broader translation to other tumor models. The insights gained from this study also deepen the understanding of structure‐function relationships in protein design, demonstrating how modest sequence‐level modifications can significantly enhance the therapeutic potential of protein‐based biomaterials. This approach has the potential to inspire innovative cancer treatments with improved therapeutic efficacy.

## 2 Results and Discussion

### 2.1 Design of Tri‐Block Chimeric cSELPs

**Figure 1:**
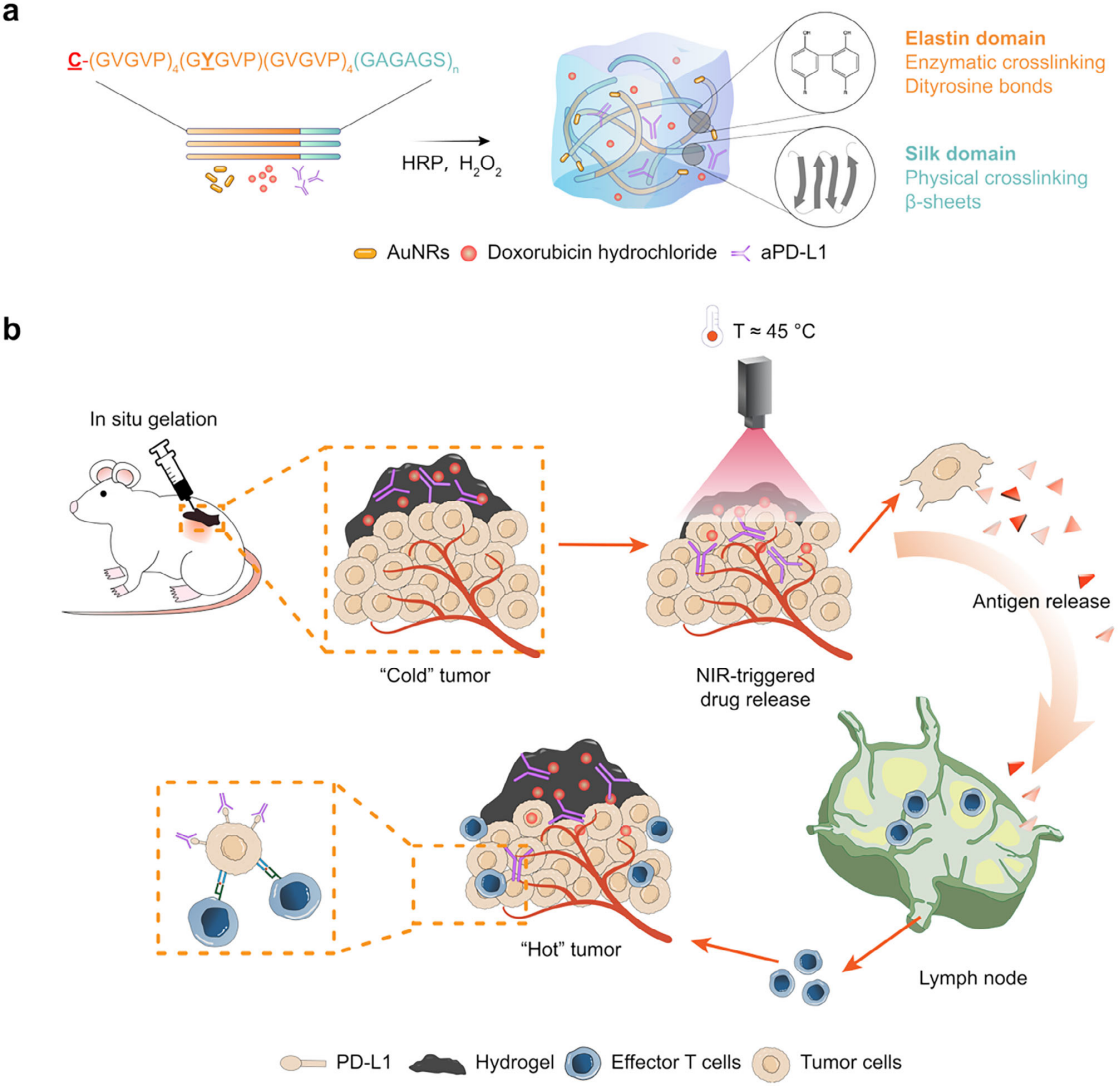
Protein design and triple‐negative breast cancer treatment strategy. a) Schematic illustration of tri‐block chimeric cSELPs forming in situ hydrogels via dityrosine bonds from elastin domains and β‐sheets from silk domains. b) Synergistic photothermal‐triggered chemo‐immunotherapy using cSELP in situ photothermal hydrogel depots. NIR‐triggered mild heat results in the release of both chemo‐ and immunotherapy drugs from the protein hydrogel. The system can directly kill tumor cells and reverse the immunosuppressive tumor microenvironment of both primary and distal tumors.

First, we designed and synthesized a family of tri‐block chimeric proteins, cSELPs, which can in situ gelling and respond to environmental stimuli to deliver the cargos DOX and monoclonal antibodies aPD‐L1 locally to the tumor (**Figure** **1**a). The cSELP sequences were designed with a photothermal agent binding motif, an elastin motif, and a silk motif. The photothermal agent binding motif was genetically engineered to include a cysteine residue in the protein affinity tag, facilitating the recombinant proteins to bind with AuNRs. Both the elastin motif and silk motif were selected from natural proteins (i.e., elastin in the extracellular matrix and *B mori*. silk fibroin) because of their excellent biocompatibility and unique physical properties.^[^ ^34^, ^40^ ^]^ The elastin motif (GVGVP/GYGVP blocks) was designed to respond to external thermal stimuli and provide the tyrosine crosslinking sites for hydrogel formation. The silk motifs (GAGAS blocks) were encoded as the physical crosslinking site to promote beta‐sheet formation for sustainable drug release and outstanding mechanical properties of the hydrogels.^[^ ^33^, ^34^, ^41^ ^]^ Moreover, cSELPs with various silk‐to‐elastin ratios were generated to obtain sequences with suitable lower critical solution temperatures (LCSTs). Based on the number of silk blocks in each cSELP monomer, the cSELPs were denoted as cY, c2Y, and c4Y (Table S1, Supporting Information). The cSELPs with 3 different silk‐to‐elastin ratios were synthesized via the synthetic biology approach as described previously^[^ ^33^, ^38^ ^]^ and subsequently purified using the inverse temperature transition cycling (ITC) method (Figure S1, Supporting Information).

This protein design enabled the SPTCI strategy to reshape the tumor immune microenvironment using cSELP in situ photo‐thermal hydrogel depots (Figure 1b). The SPTCI strategy started by initiating controlled mild heat generated by AuNRs under NIR irradiation, which elicited the structural transition of cSELP hydrogels to induce the release of two drugs. Meanwhile, mild heat^[^ ^42^ ^]^ and released anthracycline chemotherapy drugs^[^ ^43^ ^]^ induced tumor antigen release to trigger dendritic cells‐mediated cytotoxic T‐lymphocyte (CTL) response. Further, the systemic immune response could benefit the enhanced T cell infiltration in primary tumors and distal tumors. Lastly, aPD‐L1 released from hydrogel blocked the PD‐1/PD‐L1 interaction, further enhancing the therapeutic impact on primary tumors.^[^ ^9^ ^]^ These mechanistic pathways have been extensively validated in prior studies;^[^ ^44^ ^]^ our contribution lies in integrating them within a biocompatible, thermo‐responsive protein hydrogel platform that enables spatiotemporally controlled co‐delivery, thereby achieving synergistic inhibition of both primary and distant tumors while effectively remodeling the immunosuppressive tumor microenvironment. Rather than being restricted to TNBC or a specific drug combination, this modular delivery system holds broad applicability across diverse tumor types and therapeutic regimens, offering a promising foundation for the development of next‐generation localized combination therapies.

### 2.2 Surface Modification of AuNRs with cSELPs

Photothermal therapy (PTT) is a non‐invasive, safe, and localized treatment method with promising applications in cancer therapy. By laser irradiating the photothermal agents at tumor sites, localized hyperthermia of ≈45 °C could be achieved with minimal damage to normal tissues.^[^ ^45^ ^]^ This well‐controlled mild, transient heat can induce immunogenic cell death of tumor cells, promote vascular permeability, and increase the accumulation of immune cells in the tumor microenvironment,^[^ ^46^ ^]^ allowing a more efficacious immune‐adjuvant strategy for treating TNBC. Gold nanoparticles (AuNPs) are one of the most studied photothermal agents. The size and shape of AuNPs are key determinants of their optical, catalytic, surface, and photothermal properties. Spherical AuNPs with diameters of ≈15–50 nm are generally considered to have low toxicity.^[^ ^47^ ^]^ However, spherical AuNPs of similar sizes often exhibit relatively low maximum absorption peaks. Previous studies have shown that when the spherical AuNPs diameter ranges from 12 to 41 nm, the maximum absorption peak falls between 520 and 530 nm, with a gradual redshift as the particle size increases.^[^ ^48^ ^]^ The limited tunability of the plasmon wavelength maximum in spherical nanoparticles restricts their applicability in vivo.^[^ ^49^ ^]^ In contrast, AuNRs exhibit precisely tunable absorption peaks ranging from visible to near‐infrared regions by adjusting the aspect ratio,^[^ ^50^ ^]^ making AuNRs a more promising photothermal agent. AuNRs have been widely used as photothermal agents due to their high photothermal conversion efficiency in the NIR region and their ability to bind ligands containing ‐SH or ‐NH_2_ groups. However, free AuNRs tend to accumulate in the liver and spleen in vivo and thus can induce adverse effects, such as allergies^[^ ^51^ ^]^ and liver inflammation.^[^ ^52^ ^]^ Stabilizers like CTAB are cytotoxic,^[^ ^53^ ^]^ necessitating alternative biocompatible coatings to improve the biodistribution and clearance of AuNRs in vivo. Previous studies have shown that surface modifications (e.g., PEGylation) can improve the biodistribution and clearance of AuNRs.^[^ ^54^ ^]^ Here, we designed cSELPs to modify the surface of AuNRs, reducing their cytotoxicity while preserving the high photothermal conversion efficiency of AuNRs and the thermo‐responsiveness of cSELPs for PTT.

**Figure 2:**
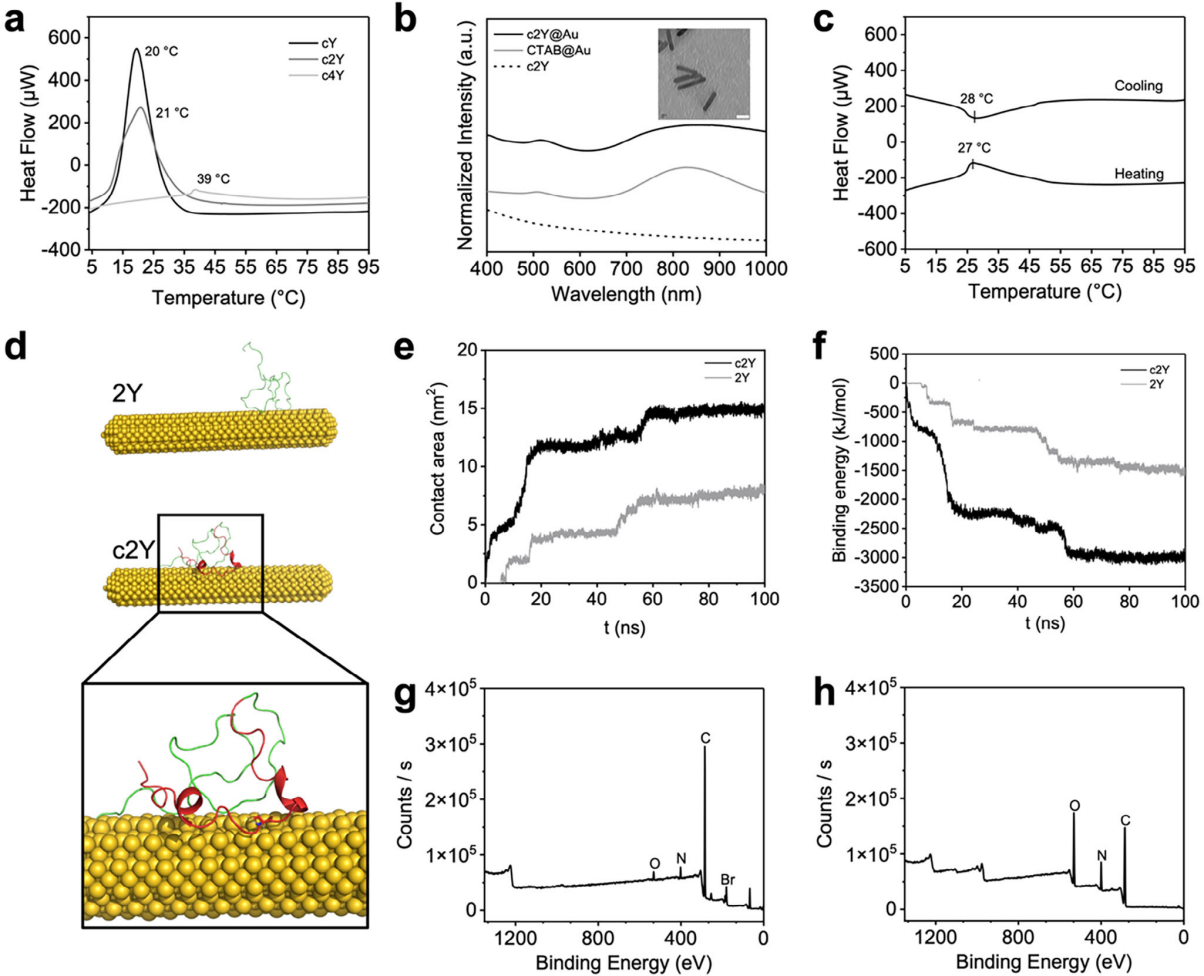
Surface modification of AuNRs with thermo‐responsive cSELPs. a) Nano DSC heat flow curves of cSELPs with different silk‐to‐elastin ratios. b) UV–vis spectrum of c2Y, CTAB‐coated AuNRs (CTAB@Au), and cSELP‐coated AuNRs (c2Y@Au) solutions. The inset shows the TEM image of synthesized AuNRs with an aspect ratio of ≈5.5 (scale bar: 40 nm). c) Heating and cooling nano DSC heat flow curves of c2Y@Au solution. d) MD simulation of the protein‐AuNR interactions at 20 ns (all water molecules and ions were hidden for display). e) Contact area and f) binding energy of c2Y‐AuNR and 2Y‐AuNR during 100 ns simulation. XPS scans of g) CTAB@Au and h) c2Y@Au hydrogels.

The thermo‐responsive properties of cSELPs were investigated using nano differential scanning calorimetry (nano DSC). The 5% w/v cY, c2Y, and c4Y protein solutions exhibited LCSTs at 20, 21, and 39 °C, respectively, when heated from 5 to 95 °C (**Figure** **2**a). The exothermic heat flow curves from the cooling process confirmed the reversible temperature transitions of cY, c2Y, and c4Y proteins (Figure S2a–c, Supporting Information). The cSELP was designed with an LCST between room temperature and physiological temperature (37 °C), allowing it to remain in a soluble or injectable state during in vitro handling. Upon exposure to physiological conditions, the material undergoes a phase transition, and the elevated temperature promotes increased drug release efficiency. In this context, cY and c2Y were selected for further experiments. The monodispersed CTAB‐coated AuNRs were synthesized by the seedless growth method.^[^ ^50^ ^]^ The obtained CTAB‐coated AuNRs possessed a rod shape with an aspect ratio of ≈5.5 (Figure 2b; Figure S3, Supporting Information). The cSELP‐coated AuNR solutions (cSELP@Au) were then developed by mixing cSELP solutions with washed AuNRs at 4 °C overnight. The UV–vis spectrum of CTAB‐coated and c2Y‐coated AuNR solutions showed prominent absorption peaks ≈800 nm, corresponding to the longitudinal surface plasmon resonance of AuNRs (Figure 2b). In addition, the resultant cY@Au and c2Y@Au solutions preserved the thermo‐responsive properties of cSELPs with LCSTs at 20 and 27 °C, respectively (Figure 2c; Figure S2d,e, Supporting Information). The LCST of c2Y@Au falls between room temperature (25 °C) and body temperature (37 °C). Therefore, we selected c2Y@Au as the drug carrier for further cancer therapy.

To understand the interaction between peptide chains and AuNRs, we conducted 100 ns molecular dynamic (MD) simulations under 1 bar and 310 K. AuNR with length and width of 10.0 and 2.3 nm, and two proteins with monomer repeat c2Y and 2Y were applied in the simulation. Both proteins were placed over 1.3 nm away from the AuNR at 0 ns and observed to gradually interact with the AuNR during the 100 ns simulation (Figure S4a, Supporting Information). The radius of gyration (Rg) values and root‐mean‐square deviation (RMSD) values of c2Y and 2Y were calculated to verify the stability of the system (Figure S4b,c, Supporting Information). During the first 20 ns, both proteins left their initial position and bound to the AuNR. The cysteine‐containing tag of c2Y was observed to quickly drag c2Y toward AuNR, thus leading to a shorter time and trajectory to reach AuNR than 2Y (Movies S1 and S2, Supporting Information). In particular, the sulfhydryl group of cysteine remained pointing to the AuNR surface (Figure 2d), which might be ascribed to the interaction between the sulfhydryl group and AuNRs. The contact area of c2Y‐AuNR and 2Y‐AuNR was also obtained during the 100 ns process. In the last 10 ns, the contact area of c2Y‐AuNR and 2Y‐AuNR was 14.9 and 7.8 nm^2^, respectively (Figure 2e). Furthermore, the stability of the protein‐AuNR binding system was evaluated by the binding energy. The binding energies of c2Y‐AuNR and 2Y‐AuNR were ‐2994.0 and ‐1486.3 kJ mol^−1^ in the last 10 ns, respectively (Figure 2f). Compared with 2Y‐AuNR, the c2Y‐AuNR system possessed a higher contact area and lower binding energy, indicating stronger binding affinity and higher binding stability. These results suggested that the designed cysteine residue in the photothermal agent binding motif could facilitate the interaction between c2Y and AuNRs, providing theoretical support for the rationality of the protein sequence design. This hypothesis was further evaluated through subsequent experimental validation.

The interaction between the gold surface and protein was also deciphered by quartz crystal microbalance with dissipation monitoring (QCM‐D) test using gold sensors. QCM‐D can monitor the frequency changes in oscillating quartz sensors, with a lower sensor oscillation frequency representing more interfacial absorption. Results suggested that the cysteine‐tagged c2Y proteins showed higher sensed mass than 2Y proteins without tags on gold sensors (Figure S5, Supporting Information). Moreover, the density of both protein absorption layers did not show significant decreases upon rinsing. QCM‐D confirmed the strong binding affinity between AuNRs and c2Y proteins in the aqueous environment. These results suggest that the cysteine‐containing tag effectively promotes the interaction between AuNRs and the protein matrix, providing experimental support for the validity of our rational sequence design.

Previous studies have shown that CTAB‐coated gold nanorods exhibit considerable cytotoxicity in both in vitro and in vivo settings due to the presence of the cationic surfactant layer.^[^ ^55^ ^]^ Therefore, thorough removal of CTAB is crucial for enabling the safe application of AuNRs in biological systems. The surface chemistry of AuNRs before and after the ligand exchange process was analyzed via X‐ray photoelectron spectroscopy (XPS) to verify the removal of CTAB. Both CTAB@Au solutions and c2Y@Au hydrogels were freeze‐dried and tested to gain the surface layer signals of the samples. As shown in the wide scan XPS spectrum, signals corresponding to carbon, nitrogen, bromine, and gold were detected in CTAB@Au samples (Figure 2g). The bromine signal was missing in c2Y@Au hydrogel samples (Figure 2h), which is one of the chemical constituents of the CTAB molecules. These results suggested the removal of CTAB from the surface of the AuNRs. Notably, the weak Au signal observed in XPS spectra was likely due to the surface sensitivity of the technique,^[^ ^56^, ^57^ ^]^ as the densely coated layer could attenuate or obscure the underlying AuNR signal (Figure 2h). Furthermore, we recorded the high‐resolution Au 4f XPS spectrum of lyophilized CTAB@Au powder and c2Y@Au hydrogels to determine the binding energies of gold elements. For CTAB@Au samples, the Au 4f_7/2_ photoelectron peak was 83.5 eV, and Au 4f_5/2_ was 87.2 eV. Also, a double peak occurred at 84.2 and 87.9 eV in c2Y@Au hydrogel samples, representing Au 4f_7/2_ and Au 4f_5/2_, respectively. These results proved the presence of the Au(0) state in both samples and the absence of the Au(I) state (Figure S6, Supporting Information).^[^ ^58^, ^59^ ^]^

The removal of CTAB was further confirmed by ^1^H nuclear magnetic resonance (NMR) spectroscopy. The proton signals of CTAB@AuNRs showed no significant changes in chemical shifts compared to pure CTAB, and the CTAB spectra were assigned by referencing published literature.^[^ ^60^ ^]^ The c2Y protein displayed chemical shift peaks for Tyr aromatic protons at 7.01 and 6.74 ppm.^[^ ^61^, ^62^ ^]^ Moreover, based on the missing characteristic peaks of CTAB in c2Y@Au, it can be concluded that CTAB was replaced by c2Y (Figure S7a, Supporting Information). The removal of CTAB was further analyzed by Fourier transform infrared spectroscopy (FTIR). By comparing the FTIR spectra of CTAB and CTAB@Au, a characteristic N–H stretching vibration peak appeared at ≈3379 cm^−1^, indicating interaction between CTAB and AuNRs through the –NH group, consistent with the formation of a bilayer structure in aqueous solution.^[^ ^63^, ^64^ ^]^ Strong C–H stretching peaks at 2914 and 2847 cm^−1^, along with a methylene‐related peak at 1461 cm^−1^, further confirmed the presence of CTAB.^[^ ^63^, ^64^ ^]^ c2Y@Au hydrogel samples did not show these characteristic absorbance peaks from CTAB, suggesting the removal of CTAB (Figure S7b, Supporting Information). Also, the FTIR absorbance at 1624 cm^−1^ proved that beta‐sheets were formed in the hydrogels after adding AuNRs into the protein matrix (Figure S7c, Supporting Information).^[^ ^33^ ^]^ Collectively, these results confirmed the removal of CTAB from the c2Y@Au system, which is anticipated to significantly enhance its biocompatibility and support its potential for in vivo applications.

### 2.3 Photothermal‐Responsive cSELP Hydrogels

**Figure 3:**
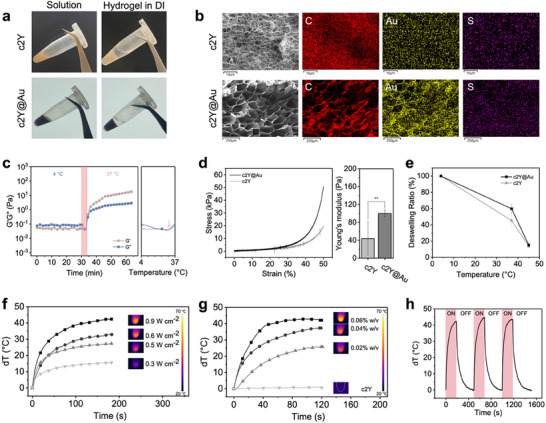
Characterization of photothermal‐responsive cSELP hydrogels. a) Optical images of the sol‐to‐gel transition of c2Y and c2Y@Au after adding HRP and H_2_O_2_. b) SEM images and element mapping of c2Y and c2Y@Au hydrogels at 25 °C. The weak Au signal seen in the c2Y mapping is likely due to background noise. c) Temperature‐dependent storage (G′) and loss (G″) modulus changes of c2Y@Au with HRP and H_2_O_2_ with constant strain of 3%. After equilibrating at 4 °C for 30 min, the temperature is rapidly increased to 37 °C at the rate of 10 °C min^−1^ and stayed at 37 °C for 30 min. d) Stress–strain curves and Young's modulus of c2Y and c2Y@Au hydrogels at room temperature. The comparison of two groups is performed using *t*‐test (^**^ *p* < 0.01). e) Deswelling profiles of c2Y and c2Y@Au hydrogels. f) Photothermal conversion property of c2Y@Au hydrogels under different laser power densities (0.9, 0.6, 0.5, and 0.3 W cm^−2^). All hydrogels are fabricated with 0.04% w/v AuNRs. g) Photothermal conversion property of c2Y@Au hydrogels with different AuNRs content (0.06, 0.04, and 0.02% w/v). All hydrogels are irradiated by 0.9 W cm^−2^ 808 nm laser for 2 min. h) Three ON (3 min) and OFF (5 min) cycles of c2Y@Au hydrogels.

c2Y is a thermo‐responsive protein polymer that exhibits LCST behavior. When the temperature rises above its LCST, hydrogen bonds between polymer chains and water molecules are disrupted, leading to enhanced polymer–polymer interactions and phase separation.^[^ ^65^ ^]^ To explore the potential of forming hydrogels based on this intrinsic thermal responsiveness, we evaluated the rheological behavior of c2Y@Au solutions at physiological temperature. At 37 °C, the c2Y@Au solution showed a slightly higher storage modulus (G′) than loss modulus (G″), which can be attributed to LCST‐induced physical association (Figure S8, Supporting Information). However, visual inspection after 1 h of incubation revealed reversible protein precipitation, which could be re‐dispersed by gentle shaking (Figure S9, Supporting Information), indicating that LCST‐driven aggregation alone was insufficient to provide long‐term mechanical stability. To address this limitation, enzymatic crosslinking was introduced to reinforce the network structure.^[^ ^33^ ^]^ This strategy enabled the formation of mechanically robust c2Y@Au hydrogels at low protein concentrations, making them suitable for further controlled drug release applications. This hypothesis was further supported by rheological measurements. When the temperature was higher than 27 °C (i.e., the LCST of c2Y@Au hydrogel precursor solutions), G′ was larger than G″ and reached a plateau state later (Figure S10, Supporting Information), indicating successful gel formation through combined thermal and enzymatic crosslinking mechanisms. Both c2Y and c2Y@Au hydrogels exhibited a porous network structure, with elemental mapping confirming the uniform distribution of gold within the c2Y@Au hydrogels (**Figure** **3**a,b; Figures S11 and S12, Supporting Information). Moreover, to mimic the in situ gelation process in vivo, the c2Y@Au hydrogel precursor solution was first equilibrated below its LCST and then increased to 37 °C at a rate of 10 °C min^−1^. The c2Y@Au hydrogel precursor solution exhibited a sol–gel transition within 3 min upon heating and remained in a gel state at 37 °C (Figure 3c). This measurement simulated the rapid temperature changes when injecting precursor solution into the body and proved that the c2Y@Au solution was competent to form injectable in situ hydrogels. To further investigate the mechanical robustness of the c2Y@Au hydrogels, strain‐sweep rheological tests were performed to evaluate their viscoelastic behavior under increasing strain (Figure S13, Supporting Information). The results showed that the storage modulus (G′) remained consistently higher than the loss modulus (G″) within the linear viscoelastic region, indicating dominant elastic behavior. At higher strains, both G′ and G″ decreased, reflecting partial disruption of the hydrogel network. These findings confirm the mechanical stability of the hydrogels under physiologically relevant stress conditions. Biomaterials with compression modulus exceeding that of breast tissue (3.25 ± 0.91 kPa^[^ ^66^ ^]^) are generally considered unsuitable for in vivo implantation. Therefore, the elastic modulus of the two hydrogels was evaluated (Figure 3d). Both hydrogels exhibited significantly lower moduli than human breast tissue, showing great potential for translational applications.

The equilibrium deswelling behavior of c2Y and c2Y@Au hydrogels was investigated under different temperatures to evaluate their responsiveness to physiological conditions. Initially, the deswelling properties were assessed in deionized water at 4, 37, and 45 °C (Figure 3e; Figure S14, Supporting Information). As the temperature increased from 4 to 37 °C, c2Y and c2Y@Au hydrogels contracted to 45% and 60% of their original volume, respectively, indicating that protein structural transitions around the lower critical solution temperature (LCST) contributed to hydrogel contraction. Further heating to 45 °C led to additional deswelling, with final swelling ratios of 14% and 15%, respectively. To better mimic physiological conditions, we further assessed the deswelling behavior of c2Y@Au hydrogels in phosphate‐buffered saline (PBS). Compared with measurements in deionized water, the hydrogel exhibited a more pronounced initial contraction, shrinking to ≈90% at 4 °C. With increasing temperature, the deswelling ratio further decreased to 30% at 37 °C and reached ≈15% at 45 °C, showing consistent temperature‐responsive behavior under physiologically relevant conditions (Figure S15, Supporting Information). SEM images of c2Y@Au hydrogel cross‐sections at 4, 37, and 45 °C (Figure S16, Supporting Information) revealed a progressive reduction in pore size in response to elevated temperatures, consistent with the macroscopic deswelling profiles observed under both water and PBS conditions.

To evaluate the PTT potential of c2Y@Au hydrogels, the nexus between temperature increases and laser power density was explored. When varying the laser power, higher power density led to a higher temperature increase (dT) after 3 min exposure (Figure 3f). The final dT was 42.4, 33.1, 27.3, and 15.7 °C, corresponding to the power density of 0.9, 0.6, 0.5, and 0.3 W cm^−2^. The results showed that the c2Y@Au hydrogels possessed superior photothermal conversion properties even in a low irradiation power (0.3–0.9 W cm^−2^), which may be ascribed to the high local concentration of AuNRs in the crosslinked hydrogels. Furthermore, we investigated the photothermal conversion ability of c2Y@Au hydrogels with varied Au concentrations (0.02, 0.04, and 0.06% w/v) using a fixed laser power of 0.9 W cm^−2^ at 808 nm at room temperature. The exact Au concentrations of the three groups were determined using ICP‐MS (Figure S17, Supporting Information). Each bar in the figure represents the mean of three independently prepared samples with the same Au content, illustrating the reproducibility of the preparation process. The temperature increased rapidly within 2 min, and the dT was positively correlated with Au concentration (Figure 3g). dT of the 0.06% w/v group was 42.9 °C, which reduced to 37.3 °C for the 0.04% w/v group and only 25.8 °C for the 0.02% w/v group. All three concentrations can induce mild PTT (45 °C) for subsequent experiments. Specifically, c2Y@Au hydrogels in the 0.06 and 0.04% w/v groups were more efficient in generating hyperthermia for direct tumor ablation than c2Y hydrogels. To ensure the mild PTT designed for in vivo treatments, c2Y@Au hydrogels with Au concentrations of 0.04% w/v and 0.9 W cm^−2^ laser irradiation powers were chosen for the subsequent experiments. Additionally, the c2Y@Au hydrogels with Au concentrations of 0.04% w/v were exposed to 0.9 W cm^−2^ NIR laser for three ON (3 min) and OFF (5 min) cycles (Figure 3h). As expected, the dT of each cycle (≈40 °C) exhibited consistent temperature increases during repeated exposures, confirming the photothermal stability of the c2Y@Au hydrogels and paving the way for the application of repeated PTT.

### 2.4 Controlled Drug Release and Antibacterial Effects of cSELP Hydrogels

**Figure 4:**
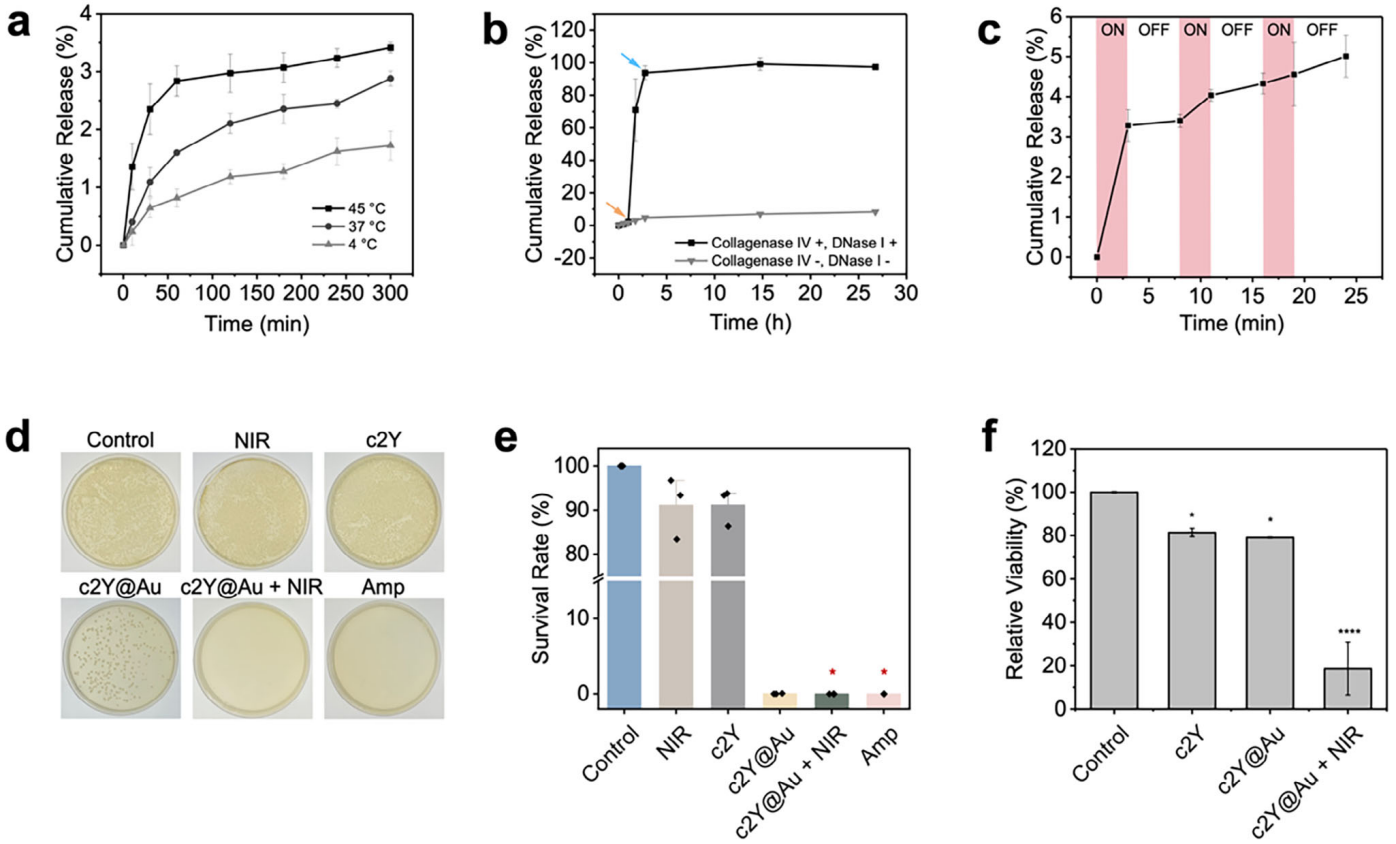
Photothermal‐responsive cSELP hydrogels with controlled drug release profile and bactericidal effects. Drug release profiles of DOX from 0.04% w/v c2Y@Au hydrogels in a) DI water at 4, 37, and 45 °C, and b) in solutions with and without high concentration digestive enzymes. The orange arrow indicates adding the digestion solution (1 mg mL^−1^ collagenase IV and 0.1 mg mL^−1^ DNase I) and transferring the samples from 4 °C to 37 °C. The blue arrow indicates the complete digestion of the bulk hydrogels. c) The NIR‐laser triggered DOX release profile of 0.04% w/v c2Y@Au hydrogels. The laser power density is 0.9 W cm^−2^. d,e) Images of bacterial colony on agar plates after being treated with Amp, NIR, c2Y hydrogel, c2Y@Au hydrogel, c2Y@Au hydrogel +NIR, and the control group without any treatment. A Kruskal–Wallis H test indicated a significant difference among the six groups (H = 16.30, df = 5, *p* < 0.01). Post hoc comparisons using Dunn's test with Holm adjustment revealed that the c2Y@Au + NIR group differed significantly from the positive control (^*^ *p* < 0.05), and the Amp group also differed significantly from the positive control (^*^ *p* < 0.05). No other pairwise comparisons reached statistical significance. f) The relative cell viability of 4T1 cells that are cultured with c2Y hydrogel, c2Y@Au hydrogel and c2Y@Au hydrogel +NIR laser. Data represent mean ± sd (n = 3 biologically independent samples). The comparison of each group with the control group is performed using One‐way ANOVA (^*^ *p* < 0.05, ^****^ *p* < 0.0001).

Next, we evaluated the drug release profiles of c2Y@Au hydrogels. Doxorubicin hydrochloride (DOX) was loaded into the hydrogel by mixing the hydrogel precursor solution with a certain amount of DOX solution. The c2Y@Au hydrogel platform achieved DOX encapsulation efficiency (EE) above 99% (Figure S18, Supporting Information), underscoring its strong capacity to retain hydrophilic drugs and its potential as an efficient delivery platform. The DOX release profiles were studied at 4, 37, and 45 °C, corresponding to the hydrogel equilibrating temperature, body temperature, and PTT treatment temperature. The c2Y@Au/DOX hydrogels exhibited a temperature‐related release behavior (**Figure** **4**a). At the 5‐h time point, the cumulative release of DOX from c2Y@Au hydrogels was 1.7% at 4 °C and increased to 2.9% and 3.4 % at 37 °C and 45 °C, respectively. Moreover, long‐term DOX release was performed at 37 °C to mimic the in vivo drug delivery process. The total release of DOX was 8.3% at 37 °C after 27 h, indicating that DOX release from c2Y@Au hydrogel was relatively slow at a constant temperature (Figure 4b). As suggested by the enzyme digestion experiment, complete degradation of the hydrogels occurred at 2.75 h (blue arrow) at 37 °C in tumor dissociation solution with a high enzyme concentration, at which the DOX cumulative release approached ≈100% (Figure 4b). These results suggested that the c2Y@Au hydrogels had superior drug retention characteristics, which might be attributed to the tightly crosslinked network and slow diffusion in the hydrogels. Notably, the enzymatic degradation solution employed here was consistent with that used for subsequent tumor dissociation experiments in immune analysis, ensuring rapid hydrogel degradation in vitro. However, in vivo TME has much lower collagenase IV concentrations, enabling prolonged hydrogel retention at the target tumor site. The sustained release property and slow‐release rate of DOX enabled the c2Y@Au hydrogels to be a temperature‐dependent drug delivery system suitable for long‐term and safe chemotherapy. Based on the temperature‐dependent release property of c2Y@Au hydrogels, we performed the NIR‐triggered release using 0.9 W cm^−2^ NIR laser for three ON/OFF cycles (Figure 4c). The hydrogel temperature during the three ON stages was maintained at ≈45 °C. Notably, a burst of DOX release was observed in the first 3 min ON stage and reached 3.3 % cumulative release, while no significant release was observed in the subsequent OFF stage. After the first cycle, the release rate was 3.4 %, about the same as the release at 45 °C for 5 h. The final release was 5.0 % at the time point of 24 min after three irradiation cycles, suggesting that the release of DOX from the hydrogel network can be enhanced via NIR external stimulation.

Collectively, the photothermal‐responsive release of DOX in c2Y@Au hydrogels was based primarily on the contraction of the hydrogel at specific temperatures, which helped accelerate diffusion and facilitate the extrusion of the drug from the hydrogel network. When the hydrogel reached the equilibrium swelling state, the subsequent drug release was mainly based on passive diffusion,^[^ ^67^ ^]^ thus maintaining a slow‐release rate and a long drug retention time. Based on the photothermal conversion capability of c2Y@Au hydrogel, external NIR light irradiation could achieve a rapid temperature change of the hydrogel, thereby inducing the hydrogel contraction and accelerating the release of the drug. This design allowed our drug delivery system to have tunable and controllable drug release profiles, which were critical in alleviating the side effects of chemotherapy drugs on the body. Furthermore, IgG was used as a substitute for aPD‐L1 to assess the release behavior of macromolecular drugs. A similar temperature‐dependent release profile was observed within 5 h (Figure S19, Supporting Information). In contrast to previously reported SELP hydrogels formed through self‐assembly, which typically required high polymer concentrations above 10% or specific sequence modifications to ensure stable drug loading,^[^ ^67^ ^]^ our cSELP hydrogels achieved an IgG encapsulation efficiency of ≈91% at a protein concentration of only 5%. Therefore, we hypothesized that the in vivo encapsulation efficiency of aPD‐L1 within the hydrogel would also approach near‐complete encapsulation. This finding underscores the enhanced encapsulation capacity and sustained release performance of our platform for delivering macromolecular drugs.

Given that surgical resection remains a primary treatment for TNBC, postoperative bacterial infections, especially at incision or implant sites, pose a significant clinical risk. In the past, the excessive and improper use of antibiotics in related treatment has led to the emergence of multidrug‐resistant bacteria,^[^ ^68^ ^]^ which has brought significant challenges and difficulties for antibacterial treatment. Therefore, developing biomaterials with intrinsic antibacterial properties could greatly enhance postsurgical recovery of TNBC and reduce dependence on antibiotics. Recently, PTT has been considered an efficient strategy to combat multidrug‐resistant bacteria.^[^ ^69^ ^]^ Therefore, we expected that the photothermal effects generated by c2Y@Au hydrogels under NIR irradiation could also show outstanding bactericidal behavior. The antibacterial capacity of hydrogels against gram‐negative Escherichia coli (E. coli) was evaluated (Figure 4d,e). After incubating on the c2Y hydrogel surfaces for 2 h or irradiating by the NIR laser for 30 min, the survival rate of E coli was 91.15 or 91.11 %, respectively. However, the survival rate significantly decreased to 0.03 % when incubating with c2Y@Au hydrogels for 2 h. No bacterial colony was observed in c2Y@Au hydrogels with 30 min NIR irradiation and ampicillin groups. These results indicated that c2Y@Au hydrogels were capable of exerting antibacterial effects even in the absence of NIR irradiation. Previous studies have shown that gold nanoparticles can exert bactericidal activity by disrupting bacterial cell membranes, binding to plasmids, inhibiting enzymatic activity, or interfering with protein synthesis.^[^ ^70^ ^]^ Therefore, we hypothesized that the strong antibacterial activity of the c2Y@Au hydrogel originated from the direct contact between bacterial cells and AuNRs within the hydrogel network. Notably, complete bacterial eradication required local heat generated by NIR laser exposure, which could induce bacteria lysis and structural disintegration,^[^ ^70^ ^]^ thereby achieving antibacterial efficacy comparable to that of conventional antibiotics.

We also evaluated the cytotoxicity of c2Y and c2Y@Au hydrogels with or without 3 min NIR laser exposure on 4T1 mice breast tumor cell line (Figure 4f). The cell viability remained at 83.7 % and 81.3 % in the presence of c2Y and c2Y@Au hydrogels without irradiation, respectively (Figure 4f). In vitro PTT was performed by exposing the cells to c2Y@Au hydrogels with 3 min 0.9 W cm^−2^ NIR irradiation. The cell viability significantly decreased to 19.2 %, showing that the photothermal effects of c2Y@Au hydrogels were cytotoxic to the 4T1 breast cancer cell line.

### 2.5 SPTCI Efficacy on Primary Tumors

**Figure 5:**
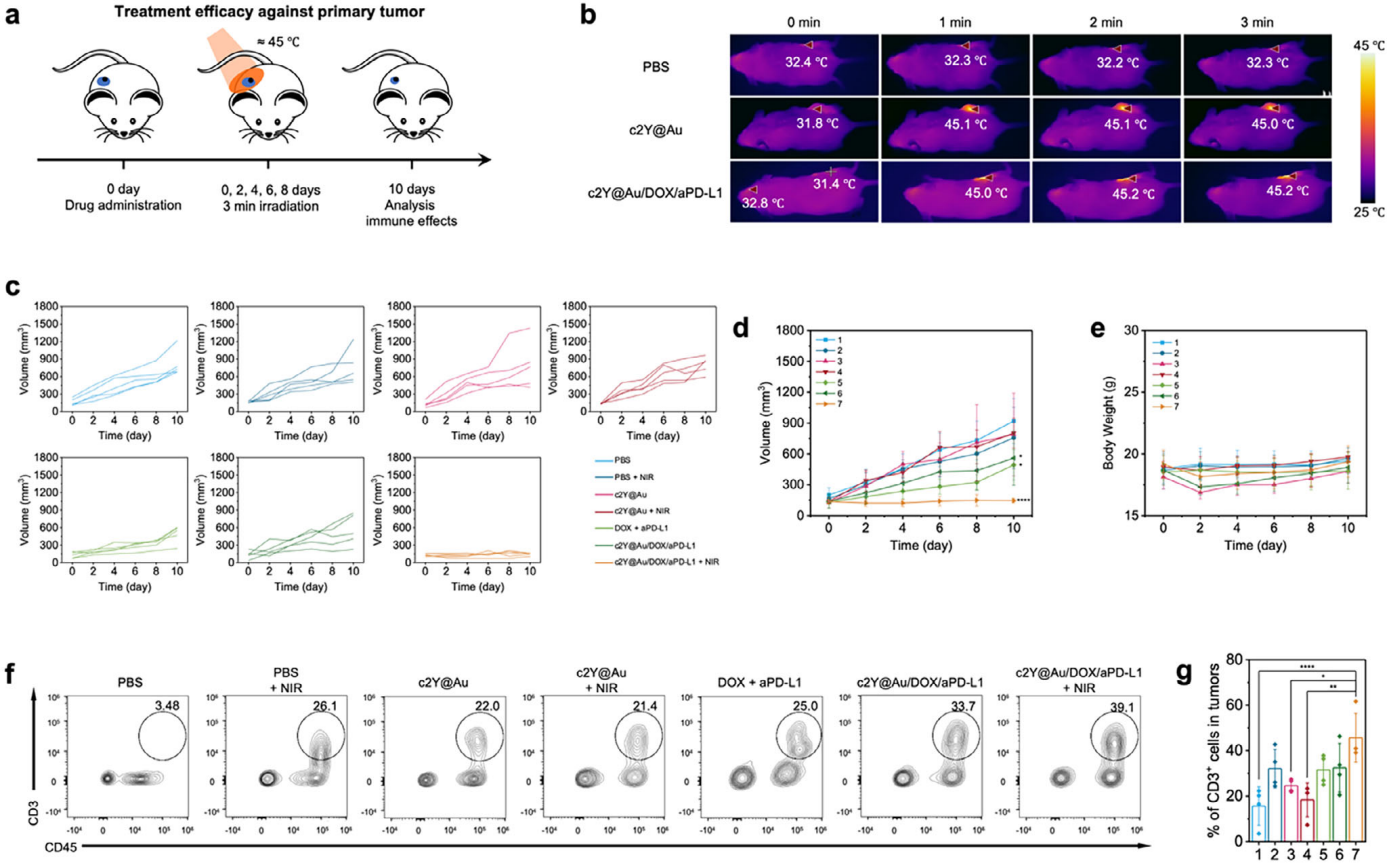
Treatment efficacy of the cSELP‐based SPTCI strategy on the primary 4T1 tumor growth. a) Schematic illustration of the single‐tumor experiment design. Black circles represent c2Y@Au hydrogels, and blue circles represent tumors. b) Infrared thermal images of tumor‐bearing mice in the PBS + NIR, c2Y@Au + NIR, and c2Y@Au/DOX/aPD‐L1 + NIR groups, recorded at 0, 1, 2, and 3 min after laser irradiation. Red arrows indicate the highest temperature in the image, while white crosses mark the tumor site temperature. c) Individual tumor growth curve of primary tumors. d) Tumor growth curves and e) body weight of single 4T1 tumor‐bearing BALB/c mice treated with PBS (1), PBS + NIR (2), c2Y@Au (3), c2Y@Au + NIR (4), DOX + aPD‐L1 (5), c2Y@Au/DOX/aPD‐L1 (6), and c2Y@Au/DOX/aPD‐L1 + NIR (7). Data represent mean ± sd (n = 5 biologically independent samples). f,g) Intratumor infiltration of T cells (CD45^+^CD3^+^). Data represent mean ± sd (n = 4 biologically independent samples). The comparison among groups is performed using One‐way ANOVA (^*^ *p* < 0.05, ^**^ *p* < 0.01, ^****^ *p* < 0.0001).

The orthotopic 4T1 tumor model of triple‐negative breast cancer was used to validate the treatment efficacy of the cSELP‐based synergistic photothermal‐triggered chemo‐immunotherapy strategy in vivo. 4T1 tumors were subcutaneously inoculated in BALB/c mice. Once the tumors grew to ≈150 mm^3^, the 4T1‐tumor‐bearing BALB/c mice were randomly divided into seven groups, including PBS, PBS + NIR, c2Y@Au hydrogels, c2Y@Au + NIR, DOX + aPD‐L1, c2Y@Au/DOX/aPD‐L1 hydrogels, and c2Y@Au/DOX/aPD‐L1 + NIR group. The dose of DOX and aPD‐L1 was 4.3 and 100 µg per mouse in the corresponding groups. The in situ c2Y@Au hydrogel underwent a rapid sol‐to‐gel transition upon injection, physically conforming to the tumor architecture and forming a stable depot that ensured local retention of therapeutics throughout the treatment period. Mice in groups requiring NIR irradiation were exposed to an 808 nm NIR laser for 3 min on days 0, 2, 4, 6, and 8 after drug administration (**Figure** **5**a,b). The therapeutic effects were evaluated by monitoring the tumor volume changes of 4T1 tumors in mice (Figure 5c,d). The PBS and c2Y@Au +NIR treated mice exhibited a similar tumor growth rate, indicating that mild PTT alone did not have a significant therapeutic effect on 4T1 tumors. This observation may be due to the restricted area of laser irradiation, which resulted in ineffective photothermal treatment of the marginal regions in the tumors. Also, previous research suggested that mild heat would cause upregulation of cell protection proteins, such as heat shock protein (HSP) and PD‐L1,^[^ ^71^ ^]^ thus enhancing the self‐protection of tumor cells. The tumor growth rates of PBS + NIR and c2Y@Au groups showed no significant difference from the PBS group, suggesting that neither the hydrogel materials nor NIR light were the main factors inhibiting tumor growth. It is worth noting that the c2Y@Au/DOX/aPD‐L1 group showed tumor inhibition to a lesser extent compared with the free DOX + aPD‐L1 group, which may be related to the strong drug retention ability of c2Y@Au hydrogels. The hydrogels limited the cumulative release of drugs within a limited time in the absence of external NIR stimulation, resulting in inferior treatment efficacy to that of the free drug group. This problem was solved by introducing NIR irradiation, which can stimulate the c2Y@Au hydrogels to increase drug release and generate mild heat to provide a TME more suitable for immune responses. This dual functionality of PTT underlies the synergistic antitumor efficacy observed in our system. The c2Y@Au/DOX/aPD‐L1 + NIR group exhibited a remarkable tumor growth inhibition of 84.2% compared to the PBS group, highlighting the exceptional therapeutic efficacy of the cSELP‐based SPTCI strategy. Moreover, the negligible difference in mouse body weight among all groups (Figure 5e) suggested that this treatment is well‐tolerated and biocompatible.

Tumor‐infiltrating T cells play a prominent role in the immune responses based on tumor cell elimination. Tumors with low levels of tumor‐infiltrating lymphocytes (TILs) possess inadequate responses toward ICB therapy, which have been defined as immunologically “cold” tumors.^[^ ^72^ ^]^ To verify the ability to turn “cold” tumors into “hot” tumors via the SPTCI strategy, we analyzed the T cells (CD45^+^CD3^+^) in tumors on day 10 after the treatments (Figure 5f,g; Figure S20, Supporting Information). Intriguingly, the c2Y@Au/DOX/aPD‐L1 + NIR group realized the highest TIL level, which was 2.9‐fold higher than that of the PBS group. Meanwhile, T cell infiltration in the tri‐modal therapy group was significantly higher than that in the c2Y@Au and c2Y@Au + NIR groups, indicating that the contribution of c2Y@Au hydrogels and PTT monotherapy to converting “cold” tumors into “hot” tumors was relatively limited. CD8^+^ T cells have been recognized as the anti‐tumor immunity paradigm, and the tumor‐infiltrating CD8^+^ T cell level plays an essential role in ICB therapy response.^[^ ^73^ ^]^ Therefore, we further analyzed the populations of CD45^+^CD3^+^CD4^+^ T cells and CD45^+^CD3^+^CD8^+^ T cells in tumors. Markedly, the c2Y@Au/DOX/aPD‐L1 + NIR group exhibited the greatest T cell infiltration and the highest proportion of CD8^+^ T cells (42.2%), ≈1.4‐fold higher than that of the PBS group (Figure S21a,b, Supporting Information). This proportion was also significantly greater than that in the c2Y@Au group and the free DOX + aPD‐L1 group, underscoring the enhanced immune activation achieved through the synergistic therapy. These findings can be explained by the lack of inherent immunotherapeutic properties in c2Y@Au hydrogels and the limited retention time of free drugs, which hindered the induction of a robust immune response. By employing c2Y@Au hydrogels as dual‐drug carriers, drug retention at the tumor site was markedly prolonged, thereby overcoming the limitations of the free drug group. Furthermore, NIR irradiation facilitated controlled burst drug release while simultaneously coordinating the synergy of the three therapeutic modalities, ultimately maximizing T cell infiltration and promoting CD8^+^ T cell levels. These results proved that the SPTCI strategy effectively induces positive synergistic effects, transforming “cold” tumors into “hot” tumors and enhancing tumor sensitivity to ICB therapy. Moreover, in the spleens, the population of CD8^+^ T cells and CD4^+^ T cells of c2Y@Au/DOX/aPD‐L1 + NIR group was both higher than other groups, which were 1.4 and 1.8 times those of the PBS group, respectively, indicating the stimulation of systemic immune response by the SPTCI strategy (Figure S21c,d, Supporting Information).

### 2.6 SPTCI Efficacy on Abscopal Tumors

**Figure 6:**
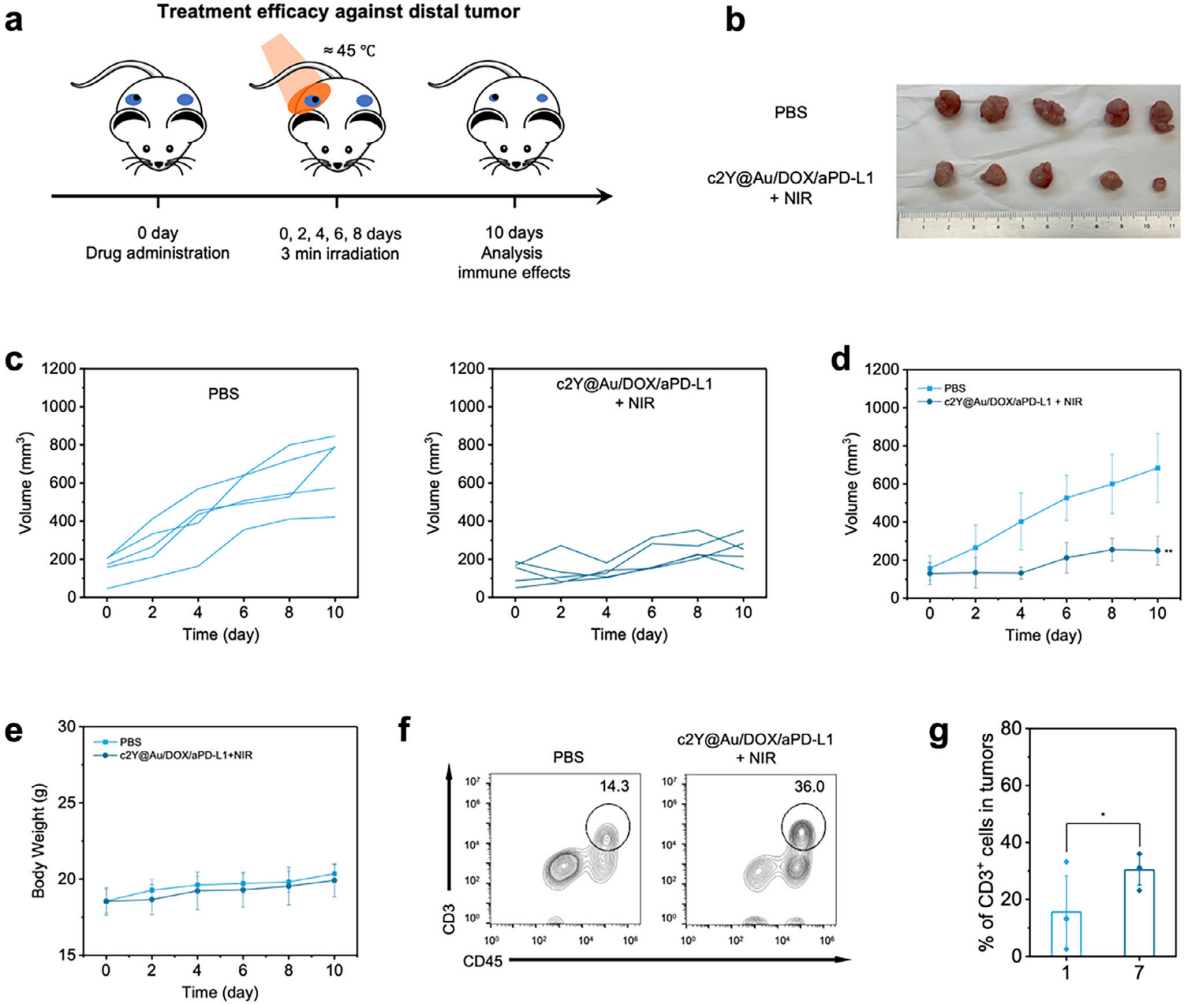
Treatment efficacy of the cSELP‐based SPTCI strategy on the distal 4T1 tumor growth. a) Schematic illustration of the two‐tumor experiment design. Black circles represent c2Y@Au hydrogels, and blue circles represent tumors. b) Image of dissected distal tumors after the treatment. c) Individual tumor growth curve of the distal tumors. d) Distal tumor growth curves and e) body weight of two 4T1 tumors‐bearing BALB/c mice treated with PBS and c2Y@Au/DOX/aPD‐L1 + NIR (n = 5 biologically independent samples). f,g) Intratumor infiltration of T cells (CD45^+^CD3^+^) treated with PBS (1) and c2Y@Au/DOX/aPD‐L1 + NIR (7). Data represent mean ± sd (n = 4 biologically independent samples). The comparison of each group with the control group is performed using *t*‐test (^*^ *p* < 0.05, ^**^ *p* < 0.01).

Inspired by the strong therapeutic effects toward primary tumors and the effective T cell immune response in the c2Y@Au/DOX/aPD‐L1 + NIR group, we further examined the potential of inhibiting abscopal tumor growth using the cSELP‐based SPTCI strategy. Mice were implanted with two tumors in both right and left flanks, and the local treatments were applied to the primary tumors once the distal tumor grew to ≈100 mm^3^ (**Figure** **6**a). Two treatment groups were designed, including PBS and c2Y@Au/DOX/aPD‐L1 + NIR groups. The size changes of the distal tumors revealed a reduced growth rate in the c2Y@Au/DOX/aPD‐L1 + NIR group, achieving a 63.5% inhibition by day 10 (Figure 6b–d). Treatment was also well tolerated, as shown by the negligible difference in the mice body weight among the two groups (Figure 6e). By analyzing TIL populations in distal tumors, a significantly higher proportion of CD3^+^ T cells was observed in c2Y@Au/DOX/aPD‐L1 + NIR group, suggesting the alleviation of immunosuppressive TME of distal tumors (Figure 6f,g). The c2Y@Au/DOX/aPD‐L1 + NIR group exhibited an ≈5‐fold increase in CD45^+^CD3^+^CD8^+^ T cells in distal tumors compared with the PBS group (Figure S22a,b, Supporting Information). In the spleen, mice treated with c2Y@Au/DOX/aPD‐L1 + NIR showed enhanced CD8^+^ and CD4^+^ T cell populations, further supporting systemic immune activation capable of suppressing distal tumor growth (Figure S22c,d, Supporting Information). These results suggest that the cSELP‐based SPTCI strategy can simultaneously treat abscopal tumors by activating a systemic immune response, while maintaining systemic tolerability and effectively suppressing orthotopic tumor growth.

### 2.7 Biosafety Evaluation

**Figure 7:**
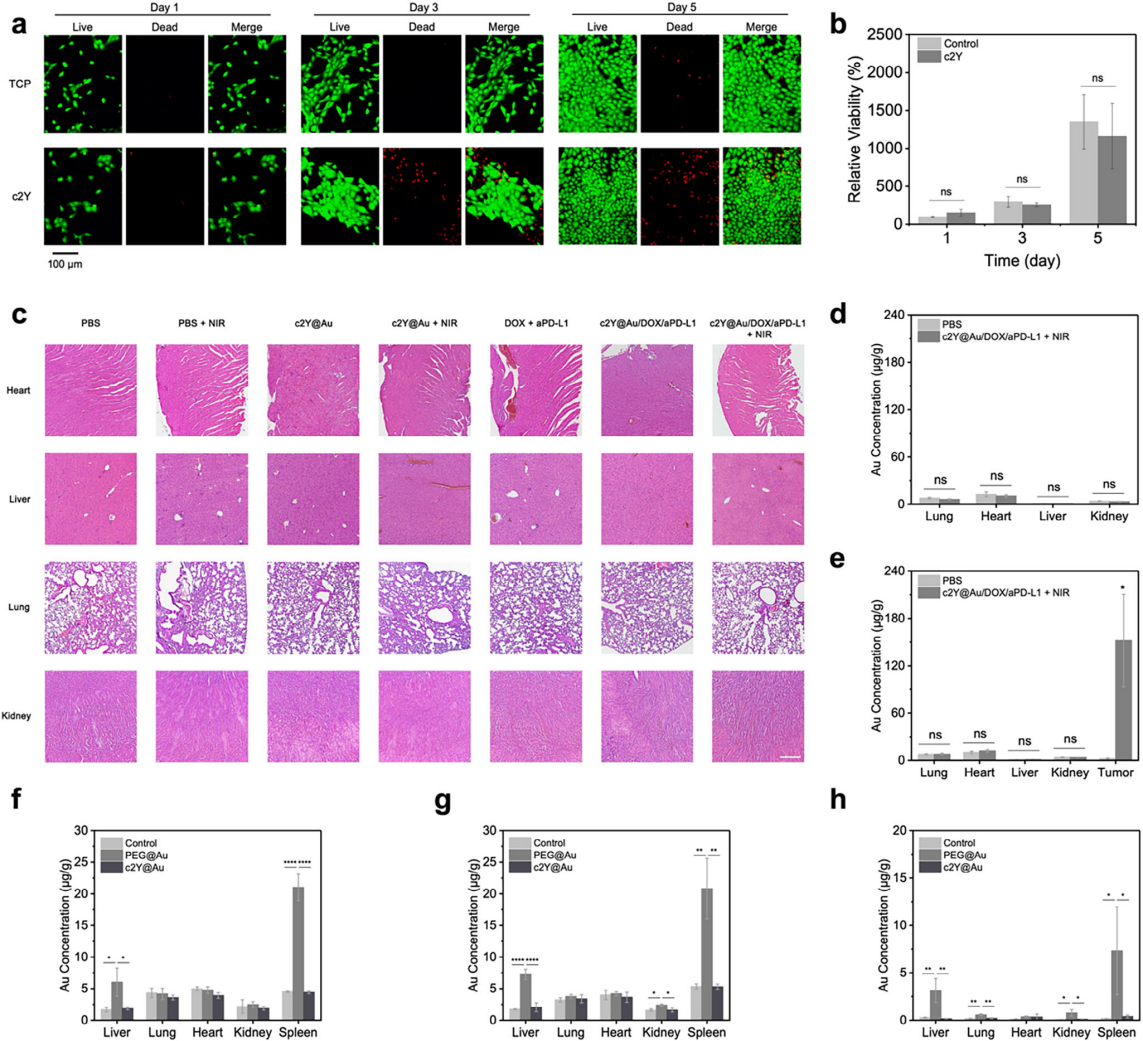
Biocompatibility of c2Y and c2Y@Au hydrogels. a) Live/dead staining and b) CCK8 results of MCF10A cells on TCP and c2Y hydrogels, showing the good biocompatibility of SELP hydrogels. c) H&E staining of histopathological images of the main organs from the single‐tumor mice model after 10‐day treatment. (scale bar: 200 µm). ICP‐MS results of the Au residual distribution in d) single‐tumor mice model and e) two‐tumor mice model. The primary tumor is used for the study. Data represent mean ± sd (n = 3 biologically independent samples). The comparison of the c2Y@Au/DOX/aPD‐L1 + NIR group with the PBS group is performed using *t*‐test (^*^ *p* < 0.05). ICP‐MS results of long‐term Au biodistribution in healthy mice subcutaneously injected with PBS (control group), PEG@Au solutions, and c2Y@Au hydrogels f) 30 days, g) 90 days, and h) 180 days after drug administration. Data represent mean ± sd (n = 3 biologically independent samples). The comparison of each group with the control group is performed using one‐way ANOVA (ns *p* > 0.05, ^*^ *p* < 0.05, ^**^ *p* < 0.01, ^***^ *p*<0.001, ^****^ *p* < 0.0001).

An ideal drug delivery system requires both good biocompatibility and low cytotoxicity. Therefore, we analyzed the biocompatibility of the c2Y hydrogels. Human breast epithelial cell line (MCF10A) cells were seeded on c2Y hydrogel surfaces, and the cytotoxicity of c2Y hydrogels was assessed by live/dead staining on MCF10A cells on days 1, 3, and 5. Fluorescent images and CCK8 results demonstrated similar cell adhesion and cell growth on the tissue culture plate (TCP) and c2Y hydrogels (**Figure** **7**a,b), suggesting that c2Y proteins have minimal cytotoxicity. In addition, no significant differences were observed between mice injected with c2Y@Au hydrogels and control mice that received PBS, based on their normal blood parameters (Figure S23, Supporting Information). The biosafety of the hydrogel‐based treatment was evaluated via H&E staining of major organs (heart, liver, lung, and kidney) from tumor‐bearing mice (Figure 7c). Tissue sections were assessed in a blinded manner and assigned a semi‐quantitative pathological score ranging from 0 (normal) to 3 (most severe). Histological analysis indicated that any pre‐existing tissue alterations associated with tumor burden were not exacerbated by the treatment, with no significant increase in toxicity, inflammatory infiltration, or hepatotoxicity, demonstrating good treatment biocompatibility (Table S2, Supporting Information). Therefore, the in vivo biocompatibility of the c2Y@Au hydrogels was further confirmed.

Material stability is a critical factor for enabling the long‐term in vivo application of c2Y@Au hydrogels. In our previous study, enzymatically crosslinked SELP hydrogels showed no significant degradation or inflammatory response after four weeks of subcutaneous implantation in rats,^[^ ^37^ ^]^ confirming that the in vivo residence time of SELP hydrogels far exceeds the 10‐day treatment window required for the SPTCI strategy in this study. Consistently, c2Y@Au hydrogels retained ≈78.7% of their initial mass after 15 days of incubation in PBS at 37 °C (Figure S24, Supporting Information), highlighting their robust structural stability. More importantly, in vivo infrared thermal imaging revealed that c2Y@Au hydrogels preserved stable photothermal conversion efficiency under NIR irradiation on day 8 (Figure S25, Supporting Information), validating their functional stability during the treatment period.

Nevertheless, both silk and elastin components are inherently biodegradable and can be broken down into small peptides and eventually into amino acids.^[^ ^74^ ^]^ These degradation products are readily absorbed or metabolized in vivo. With sufficient time, the resulting fragments can reach molecular weights below the renal clearance threshold,^[^ ^67^ ^]^ and most proteins under 70 kDa are known to be eliminated from systemic circulation via glomerular filtration in the kidneys.^[^ ^75^ ^]^ These considerations underscore the importance of investigating the in vivo clearance of AuNRs conjugated to the c2Y protein. To this end, we employed ICP‐MS to monitor the biodistribution of gold over both short‐ and long‐term time points.

The biodistribution of the Au element was evaluated using ICP‐MS for the PBS and c2Y@Au/DOX/aPD‐L1 + NIR groups after 10 days of treatment (Figure 7d,e). In the single‐tumor models, the difference in Au concentration in the lung, heart, liver, and kidney in the c2Y@Au/DOX/aPD‐L1 + NIR group was negligible compared with that in the PBS group (Figure 7d). In the two‐tumor models, Au was observed to be mainly concentrated in the primary tumors (Figure 7e). The stable Au‐S linkage between AuNRs and c2Y proteins in vivo was further proved, which minimized the side effects from AuNRs to healthy organ tissues during the treatment.

To monitor the long‐term biodistribution of AuNRs in vivo, we performed 30‐, 90‐, and 180‐day studies using healthy mice subcutaneously injected with PBS (control group), PEG@AuNRs solution (PEG@Au), and c2Y@Au hydrogels (c2Y@Au), respectively. The blood routine test showed no significant differences among all three groups at each time point, confirming the long‐term biosafety of the c2Y@Au hydrogels (Figure S26, Supporting Information). Gold concentration in main organs, including liver, lung, heart, kidney, and spleen, was measured and expressed as ng/g organ. The results suggested that the free PEG@Au group showed a significantly higher gold content in the liver and spleen 30 d after the administration (Figure 7f). However, this accumulation still remains after 90 d, showing a poor clearing efficacy through the bile ducts of free PEG@Au^[^ ^47^ ^]^ (Figure 7g). Since 90 d, we also observed that the Au content in kidneys in PEG@Au group was significantly higher than that in the other two groups, meaning that kidneys also involved in the clearance of PEG@Au (Figure 7g). After 180 d, small quantities of Au were presented in lungs in PEG@Au group, while no Au accumulation was observed in control group and c2Y@Au group (Figure 7h). The gold content accumulated in liver, spleen, lung, and kidney might cause organ toxicity and dysfunction. To determine if PEG@Au and c2Y@Au induced liver toxicity, we measured plasma AST (aspartate aminotransferase) and ALT (alanine aminotransferase) at 180 d. All the values remained normal, showing that no hepatic function damage was caused even in the PEG@Au group (Figure S27, Supporting Information). In the c2Y@Au hydrogels group, no significant gold content increase was observed in any organ at 30, 90, and 180 days after administration (Figure 7f–h). These conclusions further confirmed that the tailored c2Y proteins could significantly reduce the accumulation of free Au in main organs during long‐term treatment, greatly improve biosafety, and avoid possible organ toxicity.

## 3 Conclusion 

Inspired by earlier SELP platform,^[^ ^38^ ^]^ this study presents a more precisely controllable photothermal‐responsive system and establishes its therapeutic relevance in a challenging TNBC context.^[^ ^36^ ^]^ We designed and synthesized a family of tri‐block chimeric proteins, cSELPs, with cysteine‐containing tags to replace toxic CTAB on AuNR surfaces via strong Au─S bonds, reducing cytotoxicity while preserving high photothermal conversion efficiency. The elastin motif provided enzymatic crosslinking sites and thermo‐responsiveness to facilitate the localized formation of in situ photothermal hydrogel depots, while the silk motifs served as dual crosslinking sites for a sustained release of the drug payloads. The optimized sequence, c2Y, allowed fast hydrogel formation with suitable LCST, and c2Y@Au hydrogels served as efficient reservoirs for both small‐ and large‐molecule drugs, ensuring prolonged retention and NIR‐controlled release to minimize systemic toxicity. The c2Y@Au hydrogels also exhibited remarkable antibacterial capacity, further enhanced under NIR irradiation, extending potential applications to post‐surgical infection control and wound healing.

In this study, we combined three therapeutic modalities, mild PTT, low‐dose DOX chemotherapy, and immune checkpoint blockade, to address the challenges of TNBC. Individually, each modality has limitations. Mild PTT alone does not induce an abscopal effect, and its local therapeutic efficacy requires continuous irradiation, while it may also activate heat shock responses. DOX systemic toxicity restricts dosing, and while local low‐dose administration can reduce systemic side effects, the drug diffuses quickly, and its effects on distant tumors are limited. The efficacy of immune checkpoint blockade therapy is highly dependent on the abundance of TILs, and in advanced tumors where TIL levels are low, immunotherapy often exhibits limited effectiveness. By integrating these therapies in the c2Y@Au/DOX/aPD‐L1 system, mild PTT triggered drug release, induced tumor cell apoptosis, and promoted local immune infiltration, low‐dose DOX induced immunogenic cell death and facilitated tumor antigen release, and immune checkpoint blockade effectively activated antitumor immunity, benefiting from the enhanced immune infiltration induced by PTT and DOX. This synergistic interaction exceeds the sum of individual effects (1+1+1 > 3), converting “cold” tumors into “hot” ones and activating systemic immunity for durable suppression of both primary and distal tumors, demonstrating the superior efficacy of the c2Y@Au platform.

Overall, this c2Y@Au hydrogel platform provides a versatile tool for future biomedical applications beyond those described in this study. The hydrogel reservoir could be used to deliver various cargos, including DNA/RNA, immune cells, and immune adjuvants, or serve as antibacterial dressings to prevent surgical site infections after tumor surgery. Furthermore, genetic control of protein sequences, physical and chemical modifications on protein side chains, and incorporation of functional moieties can endow the current cSELPs platform with additional capabilities, enabling its extension to diverse cancer treatments. Beyond TNBC therapy, this cSELP library offers a rich reservoir of advanced biomaterials for precision medicine, with potential applicability to other cancer types and disease models. This work highlights the transformative potential of the cSELP hydrogel system and establishes a strategic framework for future material design and therapeutic innovation, paving the way for broader translational and clinical applications.

## 4 Experimental Section

### Chemicals and Materials

*Escherichia coli* strain BL21Star (DE3) was purchased from Shanghai Weidi Biotechnology Co., Ltd (China). Acid tetrachloroaurate (III) trihydrate (HAuCl_4_⋅3H_2_O), cetyltrimethylammonium bromide (CTAB), silver nitrate (AgNO_3_), L (+)‐ascorbic acid (AA), sodium borohydride (NaBH_4_), and (Immunoglobulin G) IgG from human serum (I4506) were purchased from Sigma–Aldrich (USA). Horseradish peroxidase (HRP) was obtained from Sangon Biotech (China). Doxorubicin hydrochloride (DOX) was purchased from Beyotime Biotechnology (China). Anti‐PD‐L1 monoclonal antibodies (Clone 10F.9G2) used in animal experiments were purchased from Bio X Cell (China). 7‐AAD Viability Staining Solution, Anti‐CD45‐V605 (Clone 30‐F11), Anti‐CD3‐FITC (Clone 17A2), Anti‐CD4‐APC (Clone GK1.5), and Anti‐CD8‐V785 (53‐6.7) antibodies were purchased from BioLegend (USA).

### Recombinant Protein cSELP Expression and Purification

cSELPs were designed and synthesized as previously described.^[^ ^33^, ^38^ ^]^ Briefly, DNA sequences encoding cSE8Y, cS2E8Y, and cS4E8Y were engineered in the bacterial expression vector, pET‐19b3, and transformed into BL21Star (DE3) (Shanghai Weidi Biotechnology Co., Ltd, China). The cSELP proteins were expressed under the T7 promoter and purified using the inverse temperature transition cycling (ITC) method. SDS‐PAGE (Biosharp, China) was used to validate the purity of cSELP proteins.

### Synthesis and Characterization of AuNRs

CTAB stabilized AuNRs were synthesized using the seedless growth method as described previously.^[^ ^50^ ^]^ Briefly, 5 mL HAuCl_4_⋅3H_2_O (1.0 mm) was mixed with 5 mL CTAB (0.2 m) at room temperature, followed by the addition of 250 µL AgNO_3_ (4.0 mm) and 8 µL HCl (12.1 m). Subsequently, 70 µL of L (+)‐ascorbic acid was added to the solution, and the solution was gently shaken. Immediately after the solution became clear, 15 µL of ice‐cold NaBH_4_ (0.01 m) was added. The solution was left overnight in a 25 °C water bath. UV–vis spectrum was used to determine the absorption peak of synthesized AuNRs, and transmission electron microscopy (TEM, JEM 1010, JEOL, Jap) was applied to observe the AuNRs morphology and aspect ratio.

### Preparation of cSELPs and c2Y@Au Solutions

To prepare cSELP solutions, 5 mg lyophilized cSELP powder was dissolved in 100 µL DI water to obtain 5% w/v protein solutions. To prepare c2Y@Au solutions, CTAB‐coated AuNRs were washed twice by centrifugation at 14 000 rpm for 20 min to thoroughly remove CTAB and subsequently mixed with the c2Y protein solution. The final protein concentration was controlled to 5% w/v. The UV–vis spectrum (Spark Microplate Reader, Tecan, Australia) of c2Y, c2Y@Au, and CTAB@Au solutions was obtained between 400 and 1000 nm. To study the thermo‐responsive properties of cSELPs and c2Y@Au, the heat flow curves were taken using a nano differential scanning calorimetry (Nano DSC, TA instrument, USA). DI water was added to the reference chamber. Each sample was equilibrated at 0 °C for 10 min and then heated from 0 to 100 °C at a rate of 2 °C min^−1^ and cooled back to 0 °C using the same rate. The baseline scans were taken using the same procedure and subtracted from the sample scans.

### Preparation of c2Y and c2Y@Au Hydrogels

c2Y and c2Y@Au hydrogels were synthesized as previously described.^[^ ^33^ ^]^ Briefly, 12 µL 40 mg mL^−1^ HRP solution was added to 200 µL 5% w/v c2Y or 200 µL 5% w/v c2Y@Au solutions. Then, 0.4 µL of 30 wt.% H_2_O_2_ solution was added to the mixture to start the cross‐linking process. The protein: HRP: H_2_O_2_ mixture was left at RT for 1 h to form stable enzymatically cross‐linked hydrogels. The inverting method was used to characterize the sol–gel transition of both c2Y and c2Y@Au hydrogels by adding a certain amount of DI water.

### Molecular Dynamic (MD) Simulation

Molecular dynamic simulations were conducted between an Au nanorod (AuNR) and two different proteins by GROMACS 2021.5 package.^[^ ^76^ ^]^ The initial structure of AuNR was obtained from the CHARMM‐GUI website^[^ ^77^ ^]^ with length and width of 10.0 and 2.3 nm containing 1685 Au atoms. The modeling of two proteins (c2Y: MGHHHHHHHHHHSSGHCDDDDKHMGAGAGSGVGVPGVGVPGVGVPGVGVPGYGVPGVGVPGVGVPGVGVPGAGAGSGAGAGS, 2Y: GVGVPGVGVPGVGVPGVGVPGYGVPGVGVPGVGVPGVGVPGAGAGSGAGAGS) was conducted by D‐I‐TASSER.^[^ ^78^ ^]^ Both protein models were parameterized by the Amberff14sb force field.^[^ ^79^ ^]^ A 14 × 14 × 14 nm^3^ cubic box was established with a central AuNR (the axial direction of the AuNR was defined as the x dimension) and two proteins at least 1.3 nm away from the AuNR. Both systems were solvated in TIP3P water. 3 Na^+^ were specifically added to c2Y system to keep electrically neutral. Energy minimization was performed using the steepest descent algorithm with a force tolerance of 500 kJ mol^−1^ nm^−1^. Moreover, periodic boundary conditions were imposed in all three directions. Each system was relaxed for 1 ns under NPT ensemble, and the position restraints of a constant of 1000 kJ mol^−1^ nm^−2^ in three directions were performed on the AuNR and heavy atoms of proteins. After completing the above steps, 100 ns NPT MD simulations were performed on both systems with position restraints performed on Au atoms. The pressure was maintained at 1 bar by the Parrinello–Rahman barostat^[^ ^80^ ^]^ in an isotropic manner, and the temperature was maintained at 310 K by the V‐rescal thermostat.^[^ ^81^ ^]^ The LINCS algorithm was performed to constrain the bond lengths of hydrogen atoms. Lennard–Jones interactions were calculated within a cutoff of 1.2 nm, and electrostatic interactions beyond 1.2 nm were treated with the particle‐mesh Ewald (PME) method with a grid spacing of 0.16 nm. UCSF ChimeraX^[^ ^82^ ^]^ was used to visualize simulation results.

### Quartz Crystal Microbalance with Dissipation Monitoring (QCM‐D) Test

The experiment was conducted on a QCM‐D instrument (Q sense E1, Biolin Scientific, USA). The Au‐coated resonator (Renlu Technology, China), operated at a fundamental frequency of 5.0 mHz was used as the substrate. The resonators were first equilibrated under DI water for ≈1000 s, and the measurements of protein‐Au interaction were initiated by switching DI water to 0.1 mg mL^−1^ c2Y or 2Y protein solution. After ≈4000 s incubation, DI water was used for the washing process.

### X‐ray Photoelectron Spectroscopy (XPS)

The XPS spectra of freeze‐dried CTAB@Au and c2Y@Au hydrogels (Au content of 0.04% w/v, determined using ICP‐MS) were obtained using an X‐ray Photoelectron Spectrometer (ESCALAB 250XI, Thermo Fisher, USA) through Al Kα (1486.7 eV) radiation. The binding energy (BE) of the core level C 1s was set at 284.8 eV and applied for spectra calibration.

### Nuclear Magnetic Resonance Spectroscopy (NMR)

The ^1^H NMR spectra (400 mHz) of CTAB, CTAB@Au, c2Y, and c2Y@Au (Au content of 0.04% w/v, determined using ICP‐MS) were performed using an NMR spectrometer (Avance NEO 400, Bruker, USA) in D_2_O.

### Fourier Transform Infrared Spectroscopy (FTIR)

The FTIR spectra of CTAB, CTAB@Au, c2Y, and c2Y@Au (Au content of 0.04% w/v, determined using ICP‐MS) were obtained using an FTIR Spectrometer (INVENIO‐S, Bruker, Germany). Each measurement was performed with a resolution of 4.0 cm^−1^ and 32 co‐added scans. The background spectra were taken before the experiments and subtracted from all sample spectra.

### Scanning Electron Microscope (SEM)

The microstructure of c2Y and c2Y@Au (Au content of 0.04% w/v, determined using ICP‐MS) hydrogel cross‐sections at different temperatures was obtained using tabletop scanning electron microscopes (EM‐30+, COXEM, Korea). Images were taken using SE2 detectors at 15 kV. The energy‐dispersive X‐ray spectroscopy (EDS)‐mapping micrographs of c2Y@Au hydrogel (4 °C) were taken using SEM microscopes (Gemini 300, Carl Zeiss AG, Germany) and a high‐throughput compact EDS detector (OXFORD Xplore, Oxford Instrument Co., Ltd., UK). The sample was sputter‐coated with platinum before the EDS mapping test.

### Mechanical Analysis

Rheology studies were performed using a Discovery Hybrid Rheometer (HR20, TA Instruments, USA) using a 40.0 mm parallel geometry and a temperature‐controllable Peltier plate Aluminum. The Au content of all the c2Y@Au samples was 0.04% w/v. To evaluate the essential role of dityrosine bonds, 1.3 mL of c2Y@Au solution without HRP and H_2_O_2_ was loaded under the fixture, and a dynamic time sweep was first conducted at an angular frequency of 10 rad s^−1^ and 3% strain for 1800 s at 37 °C. Then, 1.3 mL of c2Y@Au hydrogel precursor was mixed and loaded under the geometry. An oscillation temperature ramp of 4–37 °C was conducted at a ramp rate of 0.5 °C min^−1^, constant strain of 3%, and angular frequency of 10 rad s^−1^. To evaluate the injectable property of c2Y@Au hydrogels, a dynamic time sweep was first conducted at an angular frequency of 10 rad s^−1^ and 3% strain for 1800 s and followed with an oscillation temperature ramp of 4–37 °C at a ramp rate of 10 °C min^−1^ to simulate the rapid temperature changes after injection into the body. The temperature was then maintained at 37 °C for 1800 s to ensure the sample reached a plateau modulus. The strain sweep test of c2Y@Au hydrogels was subsequently conducted at a constant frequency of 10 rad s^−1^ at 37 °C. The young's modulus of 5% w/v c2Y and c2Y@Au hydrogels was measured using the electromechanical test systems (E42, MTS, USA).

### Deswelling Properties

300 µL c2Y and 0.04% w/v c2Y@Au hydrogels were prepared and immersed in 15 mL DI water. All the hydrogel samples were equilibrated at 4 °C overnight and weighed before deswelling studies. The hydrogels were further equilibrated at 37 and 45 °C in DI water to study the stimuli‐responsive deswelling properties. The deswelling ratio was calculated as the ratio of the weight of the hydrogels under specific temperatures to the weight of the hydrogels at 4 °C. 150 µL 0.04% w/v c2Y@Au hydrogels were prepared and immersed in 15 mL PBS. Samples were weighted and then equilibrated at 4 °C. The hydrogels were further equilibrated at 37 and 45 °C to study the stimuli‐responsive deswelling properties. The deswelling ratio was calculated as the ratio of the weight of the hydrogels under specific temperatures to the initial weight of the hydrogels.

### Photothermal Conversion Studies

The photothermal conversion studies were conducted using an 808 nm NIR laser (MDL‐XF‐808, Changchun New Industries Optoelectronics Tech. Co., Ltd, China). 3 groups of 30 µL c2Y@Au hydrogels with different AuNRs content were prepared in 1.5 mL tubes, and the exact Au concentration was determined using ICP‐MS (NexlON 1000G, PerkinElmer, USA). The temperature change of the hydrogels was recorded by a thermal infrared camera (FOTRIC 224s, FOTRIC, China) from the side of the tubes.

### DOX Release from c2Y@Au Hydrogels

The 0.04% w/v c2Y@Au hydrogels were prepared as previously described with 20 µL DOX (1 mg mL^−1^). Then, the mixture was added into 1.5 mL tubes, with each tube containing 30 µL of precursor solution (Au concentration ≈ 0.04% w/v). After gelation, 1.5 mL of DI water was used as the DOX‐release medium. All the samples were divided into four groups (4, 37, 45 °C, and NIR‐triggered) and stabilized at 4 °C for 1 h before being transferred to experimental temperatures. For the NIR‐triggered group, each sample was irradiated with an 808 nm laser at a power density of 0.9 W cm^−2^ for 3 cycles (3 min ON and 5 min OFF). At the desired interval, 200 µL of the medium was collected and substituted with 200 µL of fresh DI water. Additional samples were prepared for the control group and enzyme digestion group to test the long‐term release profile. After adding DOX‐release medium, both groups were stabilized at 4 °C for 1 h and then incubated at 37 °C. Specifically, a 1.5 mL enzyme mixture (1 mg mL^−1^ collagenase IV and 0.1 mg mL^−1^ DNase I) was used as the release medium for the enzyme digestion group, and 10 µL of the medium was collected at each time point. The aliquots collected from the enzyme digestion group were diluted 10 times before measurement. c2Y@Au hydrogel without DOX was enzymatically digested under the same conditions, and the fluorescence intensity after ten‐fold dilution was set as the background and deducted. The released amount of DOX was measured by fluorescence (λ_ex_ = 470 nm; λ_em_ = 585 nm). Diluted free DOX was used as a standard curve. Approximately 2.6, 5.2, 10.3, and 15.5 µg of DOX were respectively loaded into 30 µL of c2Y@Au hydrogel. EE was calculated as: EE (%) = (total drug added − free, non‐entrapped drug) ÷ total drug added.

### IgG Release from c2Y@Au Hydrogels

30 µL 0.04% w/v c2Y@Au/IgG hydrogel (60 µg IgG) was added into each 1.5 mL tube as the experimental group, while 30 µL c2Y@Au hydrogels were prepared as the control group. 1.5 mL of DI water was used as the IgG‐release medium. Both groups were stabilized at 4 °C for 1 h before being incubated at experimental temperatures (4, 37, and 45 °C). At the designed interval, 20 µL of the medium was collected from each sample, and 20 µL of fresh DI water was subsequently added. The release amount of protein was measured using a BCA protein assay kit (Solabio, China). Diluted free IgG was used as a standard curve. At each time point, the average protein released amount of the control group was considered as c2Y protein degradation. The IgG release amount was calculated by subtracting the c2Y amount from the amount of protein released in each sample of the experimental group.

### Antibacterial Efficacy

E. coil (ATCC 25922) was applied to test the antibacterial properties of the hydrogels. In brief, six groups were designed for different treatments, including (1) positive control, (2) 0.1 mg mL^−1^ Ampicillin sodium (Sangon biotech), (3) 808 nm NIR laser, (4) c2Y hydrogels, (5) 0.04% w/v c2Y@Au hydrogels, and (6) 0.04% w/v c2Y@Au + NIR. The study was performed on 96‐well plates. For groups (4)–(6), each well contained 80 µL c2Y or c2Y@Au hydrogel, and then 10 µL bacterial suspension (in PBS, 10^6^ CFU mL^−1^) was added onto the surface of the hydrogel. Then, for groups (3) and (6), 0.9 W cm^−2^ 808 nm NIR laser was applied to irradiate the corresponding well from the bottom of the 96‐well plate for 30 min. Additionally, sterilized PBS was added to the plate as the negative control to ensure that the experiment was free from contamination. After incubating the 96‐well plate at 37 °C for 2 h, hydrogel and residual bacterial solution in each well were collected in 1 mL of sterilized PBS and vortexed for at least 90 s to resuspend bacterial survivors. The collected bacterial solutions were diluted to an appropriate concentration and plated onto 10 cm LB agar plates. The colony‐forming units (CFU) were counted after incubating at 37 °C for 18 h.

### In Vitro PTT

The 4T1 cell line was purchased from the American Type Culture Collection (ATCC). 4T1 cell lines were cultured in RPMI 1640 medium with L‐Glutamine (Biological Industries, USA), supplemented with 10% fetal bovine serum (ExcellBio, China) and 1% antibiotic/antimycotic (Thermofisher, China). 4T1 cells were seeded in a 96‐well plate at a density of 1 × 10^4^ cells per well and cultured at 37 °C and 5% CO_2_ one day before the study. 50 µL c2Y hydrogels and 50 µL 0.04% w/v c2Y@Au hydrogels were added to the wells. Cells with hydrogels were divided into three groups (c2Y group, c2Y@Au group, and c2Y@Au + NIR group), while cells without hydrogels were used as controls. For the c2Y@Au + NIR group, cells were exposed to an 808 nm laser at 0.9 W cm^−2^ for 3 min. After the laser irradiation, all hydrogels were removed, and the relative cell viability was measured using a CCK8 cell counting kit (TransDetect, China).

### Single‐Tumor Model

BALB/c mice (6–8 weeks old, 18–20 g) were purchased from the Charles River Laboratories (Zhejiang, China) and were housed in the Zhejiang University Laboratory Animal Center. All experimental designs and protocols were approved by the Zhejiang University Ethics Committee (Ethics Code. ZJU20230009). 4T1 cells (1 × 10^6^) suspended in PBS and Matrigel (0827245, ABW, China) at a volume ratio of 1:1 were subcutaneously injected into the right flank of female BALB/C mice. The treatment started when the mean tumor volumes reached 150 mm^3^. The 4T1 tumor‐bearing mice were divided into seven groups randomly and administrated with 50 µL of PBS, PBS + NIR, c2Y@Au, c2Y@Au + NIR, free DOX + aPD‐L1 solution, c2Y@Au/DOX/aPD‐L1, and c2Y@Au/DOX/aPD‐L1 + NIR. The Au content of all the c2Y@Au samples was 0.04% w/v. Mice weights and tumor volumes were measured every other day with a vernier caliper, and the tumor volumes were calculated as width^2^ × length × 0.5. For NIR treatments, an 808 nm NIR laser was applied for 3 min every other day for five irradiations. On day 10, mice were euthanized for analysis. Specifically, primary tumors and spleens were resected from the mice, and their splenic and tumor‐infiltrating lymphocytes were analyzed using flow cytometry (Aurora Flow Cytometry, Cytek, USA). For the spleen samples, 7‐AAD, anti‐CD4‐APC, and anti‐CD8‐V785 antibodies were used according to the standard protocols, while tumor samples were incubated with 7‐AAD, anti‐CD45‐V605, anti‐CD3‐FITC, anti‐CD4‐APC, and anti‐CD8‐V785 antibodies.

### Two‐Tumor Model

To evaluate the abscopal therapeutic effect, a two‐tumor mice model was established by subcutaneously injecting 4T1 cells (1 × 10^6^ cells per site) into both the left and right flanks of each female mouse. After the distal tumor volume reached ≈100 mm^3^, all mice were divided into two groups randomly. 50 µL PBS or c2Y@Au/DOX/aPD‐L1 + NIR were administrated to the tumors at the right flanks and subsequently treated as described above. The Au content of all the c2Y@Au samples was 0.04% w/v. The mice weights and distal tumor volumes were recorded. The spleens and distal tumors were obtained on day 10 for further immune effect evaluation.

### Biocompatibility Evaluation

The MCF10A cell line was purchased from the American Type Culture Collection (ATCC). The cells were authenticated by Sangon Biotech by short tandem repeats and were routinely checked for mycoplasma contamination using Myco‐Blue Mycoplasma Detector (Vazyme, China). MCF10A cell lines were cultured in DMEM F‐12 (Shanghai BasalMedia, China) with Glutamax (Gibco, USA) at 37 °C with 5% CO_2_. The media was supplemented with 5% horse serum (Biological Industries, USA), 20 ng mL EGF (Gibco, USA), 0.5 mg mL Hydrocortisone (MedChemExpress, China), 100 ng mL Cholera toxin (Sigma–Aldrich, China), 10 µg mL Insulin (Biological Industries, USA), and 1% Pen/Strep (Biological Industries, USA). Before cell seeding, 40 µL c2Y hydrogel precursor solution was added into each well on a 48‐well plate and allowed to cure for 1 h at RT. After gelation, PBS was used to completely remove residual reactants from hydrogels. Before experiments, hydrogels were incubated overnight in cell culture media at 37 °C and 5% CO_2_. Then, MCF10A was seeded at a density of 8000 cells per well and incubated at 37 °C and 5% CO_2_. The live/dead staining assay was performed using calcein‐AM/propidium iodide (Beyotime, China) for 30 min and observed by a fluorescence microscope. The relative cell viability was measured using a CCK8 cell counting kit (TransDetect, China).

Healthy mice (10–11 weeks) were used to evaluate the in vivo biocompatibility of 0.04% w/v c2Y@Au hydrogels. Blood from control mice or mice subcutaneously injected with c2Y@Au hydrogels was sampled on day 10 after the drug administration to evaluate the biosafety of the hydrogels. All samples were analyzed via Paixide Pet Hospital Paiweite Branch (Zhejiang, China) using an automatic hematology analyzer (BC‐2800 Vet, Mindray, China).

### In Vitro Hydrogel Degradation

150 µL 0.04% w/v c2Y@Au hydrogels were prepared and immersed in 15 mL PBS. All the hydrogel samples were equilibrated at 37 °C overnight and weighed before the degradation studies (day 0). All the samples were placed at 37 °C and weighted on days 3, 6, 9, 12, 15. The weight ratio was calculated as the ratio of the weight at each time point to the weight of the hydrogels on day 0.

### Treatment Safety Evaluation

The main organs, including the heart, liver, lung, and kidney, from single‐tumor model experiments were collected to perform H&E staining and ICP‐MS analyses. For objective assessment, H&E slides were evaluated in a blinded manner by an experienced pathologist from Wuhan Pinofei Biotechnology Co., Ltd., China. A semi‐quantitative scoring system was applied to assess inflammation and tissue damage: 0 = none (normal cellular morphology and tissue architecture); 1 = mild (minimal cellular morphological alterations or slight structural abnormalities); 2 = moderate (evident pathological changes with potential impact on tissue function); 3 = severe (pronounced cellular damage with marked structural disruption, potentially compromising organ function). Each group included n = 3 biologically independent animals. Also, the same organs and the primary tumors from the two‐tumor model experiments were dissected for ICP‐MS.

### Long‐Term Au Biodistribution

36 healthy BALB/c mice (6–8 weeks old, 18–20 g) were randomly divided into 9 groups, 3 groups (control, PEG@Au, and 0.04% w/v c2Y@Au) for each time point (30, 90, and 180 days). PBS (control), mPEG‐SH (MW 2000 Da, Aladdin) coated AuNRs (PEG@Au), and 0.04% w/v c2Y@Au were subcutaneously injected into healthy mice. At each time point, blood samples were collected and analyzed via the blood routine of Paixide Pet Hospital Paiweite Branch (Zhejiang, China) using an automatic hematology analyzer (BC‐2800 Vet, Mindray, China). Meanwhile, the main organs, including the heart, liver, spleen, lung, and kidney, were lyophilized and dissolved in aqua regia to evaluate the Au content using ICP‐MS. Specifically, blood plasma was collected to evaluate the AST (aspartate aminotransferase) and ALT (alanine aminotransferase) levels at 180 d. The evaluation was completed by Paixide Pet Hospital Paiweite Branch (Zhejiang, China) using a dry chemistry analyzer (DRI‐CHEM NX500, Fujifilm, Japan).

### Statistical Analysis

Unless otherwise specified, all experiments were performed with a sample size of n = 3, and data were presented as mean ± standard deviation. Differences between groups were evaluated using one‐way ANOVA, *t*‐test, or Kruskal–Wallis H test in GraphPad Instant software (GraphPad Software). Statistical significance is indicated as ^*^ *p* < 0.05, ^**^ *p* < 0.01, ^***^ *p* < 0.001, and ^****^ *p* < 0.0001.

## Conflict of Interest

The authors declare no conflict of interest.

## Author Contributions

W.H. conceived the idea and designed the experiments. X.S. led and performed most of the experiments. J.Z. participated in material synthesis and animal experiments. J.O. participated in material characterization. Z.B. participated in antibacterial experiments. T.L. participated in histology experiments. KY.Z. participated in animal experiments. KX.Z. participated in immunology data analysis. C.W. participated in animal tumor model establishment. Q.Z. participated in histology experiments. L.L. and J.W. participated in immunology analysis.

## Declaration of Generative AI and AI‐Assisted Technologies in the Writing Process

During the preparation of this work, the authors used Grammarly in order to improve the readability and language of the work. After using this tool/service, the authors reviewed and edited the content as needed and take full responsibility for the content of the publication.

## Supporting information



Supporting Information

Supplemental Movie 1

Supplemental Movie 2

## Data Availability

The data that support the findings of this study are available from the corresponding author upon reasonable request.
