# Supplementary material for: Genetically Engineered Light‐Responsive In Situ Hydrogels for Immunomodulation and Multimodal Therapy in Metastatic Triple‐Negative Breast Cancer
Source: Adv Sci (Weinh). 2025 Oct 29;13(3):e12355. doi: 10.1002/advs.202512355 (PMC12806481; doi:10.1002/advs.202512355)
Supplement: Supplementary file 1 — Supporting Information [file ADVS-13-e12355-s003.pdf]

Supporting Information

Title

Genetically Engineered Light-Responsive In Situ Hydrogels for Immunomodulation and Multimodal Therapy in Metastatic Triple-Negative Breast Cancer

Xinchen Shen[1,2], Jiajia Zhang[1], Junhan Ou[1], Ziheng Bai[1,2], Tongyan Liu[1], Kaiyue Zhang[1], Kaixiang Zhu[3], James Q. Wang[1,2], Chaochen Wang[1,2], Qianting Zhang[1,2], Linrong Lu[1], Wenwen Huang[1,2,4,5,6,7]*

[1] The Zhejiang University-University of Edinburgh Institute, Zhejiang University School of Medicine, Zhejiang University, Hangzhou 310058, China
[2] Deanery of Biomedical Sciences, Edinburgh Medical School, College of Medicine and Veterinary Medicine, The University of Edinburgh, Edinburgh EH89AG, UK
[3] Department of Cardiology of the Second Affiliated Hospital, Zhejiang University School of Medicine, Zhejiang University, Hangzhou 310058, China
[4] Department of Orthopedics of the Second Affiliated Hospital, Zhejiang University School of Medicine, Zhejiang University, Hangzhou 310058, China
[5] Dr. Li Dak Sum & Yip Yio Chin Center for Stem Cells and Regenerative Medicine, Zhejiang University School of Medicine, Zhejiang University, Hangzhou 310058, China
[6] State Key Laboratory of Biobased Transportation Fuel Technology, Zhejiang University, Hangzhou 310027, China
[7] Biomedical and Health Translational Research Centre of Zhejiang Province, Zhejiang University, Hangzhou 310003, China

*Corresponding authors

E-mail: wenwenhuang@intl.zju.edu.cn

Table S1. Sequence design of cSELPs and SELP without affinity tag.

Name	Sequence
cY	MGHHHHHHHHHHHSSGH**C**DDDDKHMGAGAGS [(GVGVP)$_3$(GYGVP)(GVGVP)$_4$ (GAGAGS)]$_{14}$
c2Y	MGHHHHHHHHHHHSSGH**C**DDDDKHMGAGAGS [(GVGVP)$_3$(GYGVP)(GVGVP)$_4$ (GAGAGS)$_2$]$_{12}$
c4Y	MGHHHHHHHHHHHSSGH**C**DDDDKHMGAGAGS [(GVGVP)$_3$(GYGVP)(GVGVP)$_4$ (GAGAGS)$_4$]$_9$
2Y	[(GVGVP)$_3$(GYGVP)(GVGVP)$_4$ (GAGAGS)$_2$]$_{12}$

Table S2. Semi-quantitative blinded pathological scoring of major organs from tumor-bearing mice following hydrogel-based treatment.

Group name	Organ	Score
Heart	PBS	0, 0, 0
	PBS + NIR	0, 0, 0
	c2Y@Au	0, 0, 0
	c2Y@Au + NIR	0, 0, 0
	DOX + aPD-L1	0, 0, 0
	c2Y@Au/DOX/aPD-L1	0, 0, 0
	c2Y@Au/DOX/aPD-L1 + NIR	0, 0, 0
Liver	PBS	1, 1, 1
	PBS + NIR	1, 1, 2
	c2Y@Au	1, 1, 2
	c2Y@Au + NIR	2, 1, 2
	DOX + aPD-L1	1, 1, 2
	c2Y@Au/DOX/aPD-L1	1, 1, 1
	c2Y@Au/DOX/aPD-L1 + NIR	2, 1, 1
Lung	PBS	1, 2, 2
	PBS + NIR	1, 2, 2
	c2Y@Au	2, 2, 1
	c2Y@Au + NIR	2, 2, 1
	DOX + aPD-L1	2, 2, 1
	c2Y@Au/DOX/aPD-L1	2, 2, 1
	c2Y@Au/DOX/aPD-L1 + NIR	1, 2, 1
Kidney	PBS	1, 0 ,1
	PBS + NIR	0, 1, 1
	c2Y@Au	1, 0, 0
	c2Y@Au + NIR	1, 0, 0
	DOX + aPD-L1	0, 0, 0
	c2Y@Au/DOX/aPD-L1	0, 0, 1
	c2Y@Au/DOX/aPD-L1 + NIR	1, 0, 0

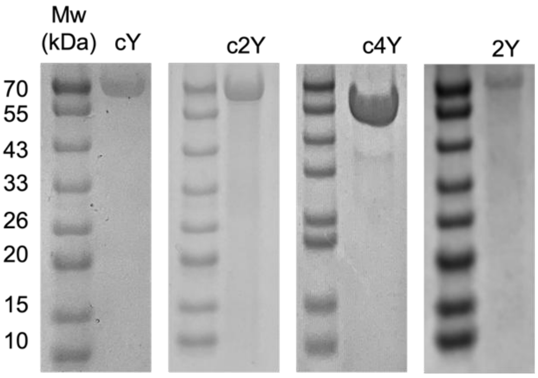

Figure S1. SDS-PAGE images of cSELPs and SELP without affinity tag.

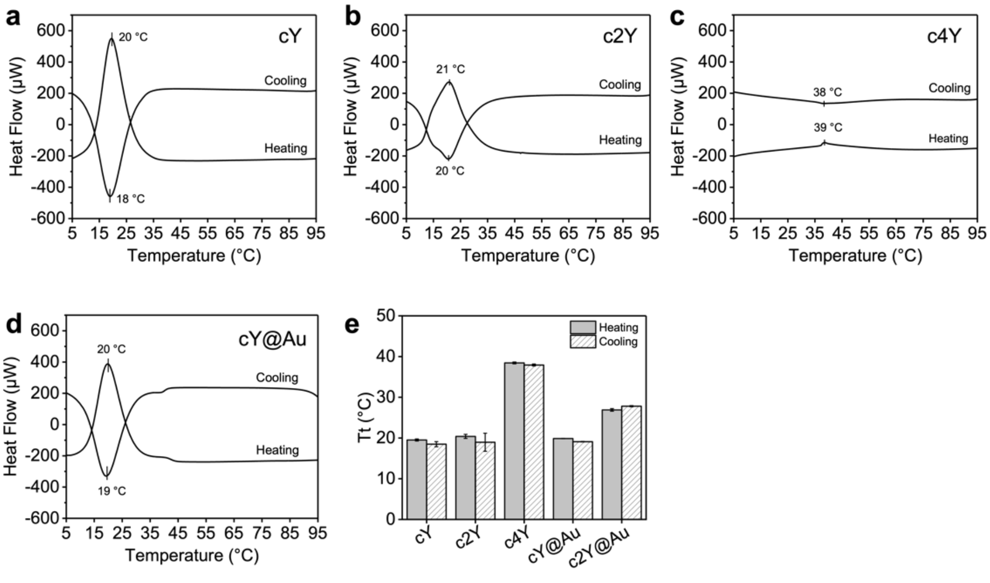

Figure S2. Heating and cooling nano DSC heat flow curves of a) cY, b) c2Y, c) c4Y, and d) cY@Au. e) The average Tt of cY, c2Y, c4Y, cY@Au and c2Y@Au under three heating and cooling cycles.

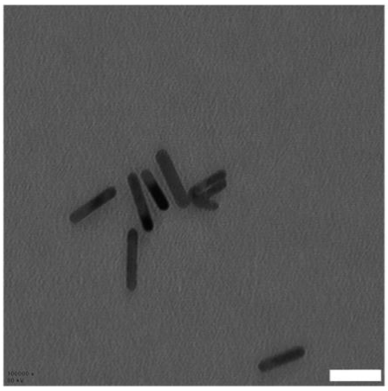

Figure S3. TEM image of CTAB@AuNRs. Scale bar: 40 nm.

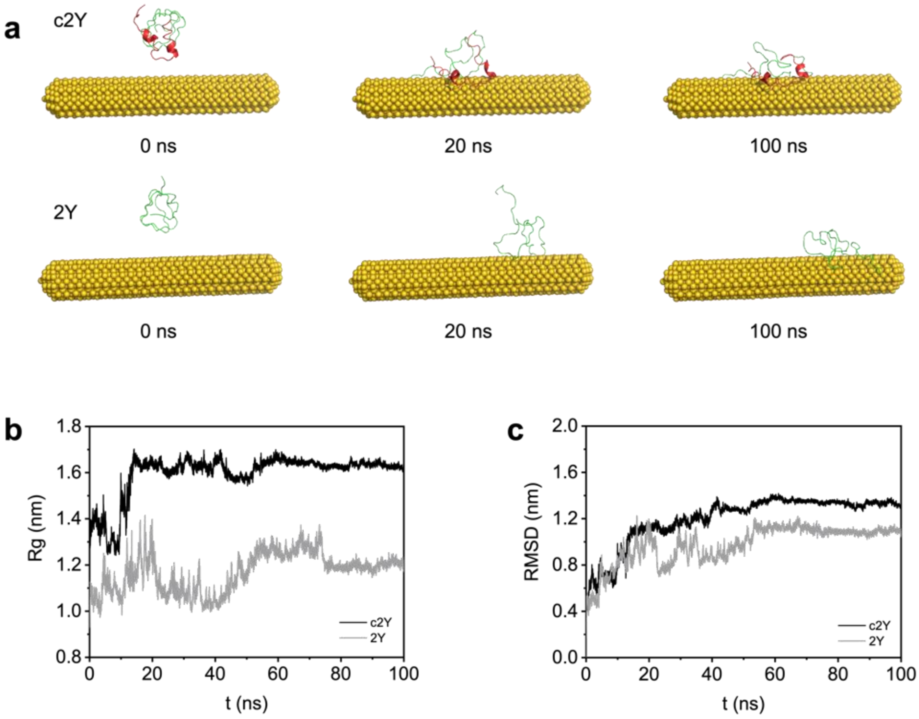

Figure S4. MD simulation of c2Y-AuNR and 2Y-AuNR interactions. a) Images of both systems at 0, 20, and 100 ns (all water molecules and ions were hidden for display). b) Radius of gyration (Rg) values of both proteins during 100 ns simulation. c) Root-mean-square deviation (RMSD) values of both proteins during 100 ns simulation.

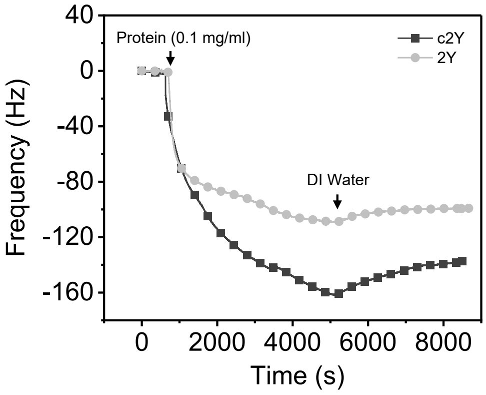

Figure S5. QCM-D test of c2Y and 2Y, suggesting the stronger protein-gold surface interaction for cSELPs.

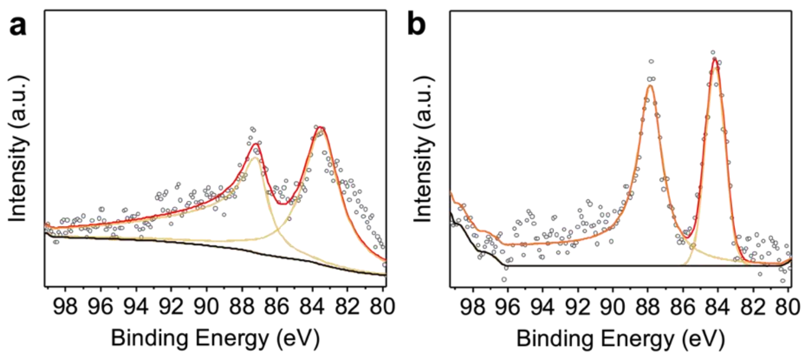

Figure S6. High-resolution Au 4f XPS spectrum of AuNRs coated with a) CTAB, and b) c2Y.

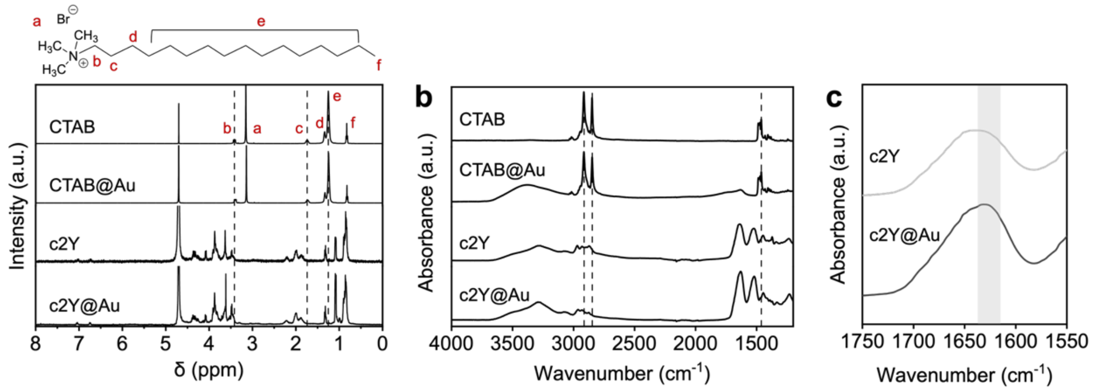

Figure S7. a) NMR spectroscopy of CTAB, c2Y, and AuNRs with different coating agents in D_2O. b, c) FTIR spectrum of CTAB, c2Y, and AuNRs with different coating agents.

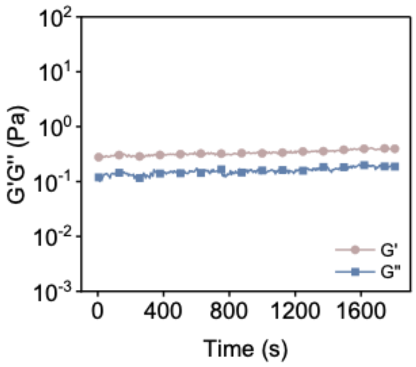

Figure S8. A dynamic time sweep of c2Y@Au without HRP and H_2O_2 at an angular frequency of 10 rad s^{-1}, 3% strain, and 37 °C.

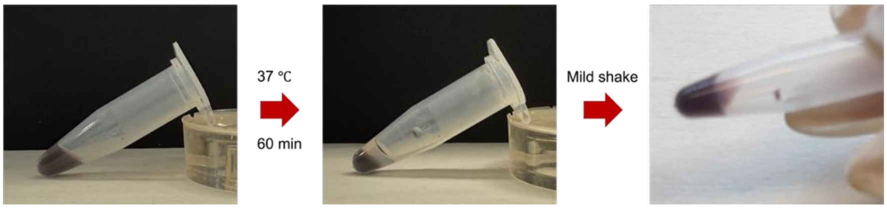

Figure S9. Optical images of the coacervation of c2Y@Au without adding HRP and H_2O_2.

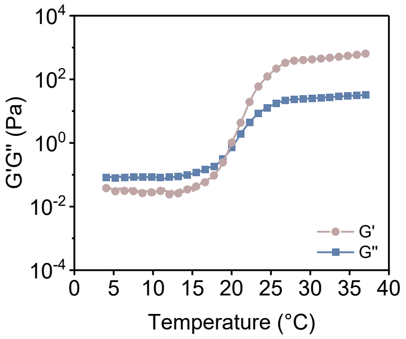

Figure S10. Storage (G′) and loss (G″) modulus changes of c2Y@Au with HRP and H_2O_2. The temperature increasing rate was 0.5 °C/min.

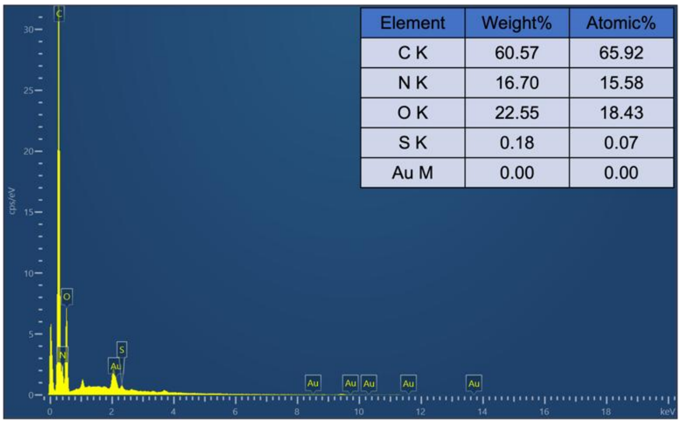

Figure S11. The element table of the EDS mapping of c2Y hydrogel.

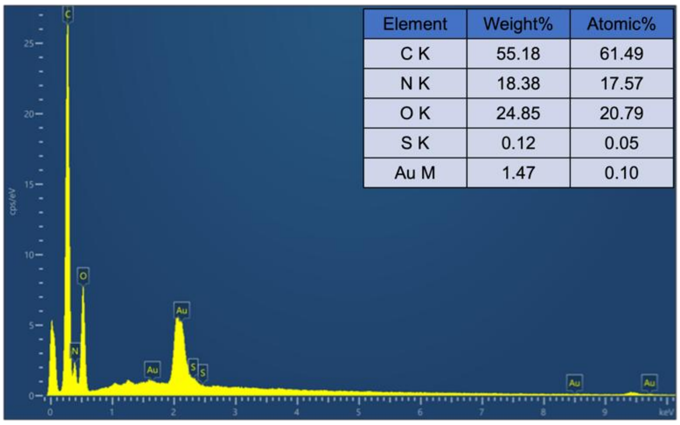

Figure S12. The element table of the EDS mapping of c2Y@Au hydrogel.

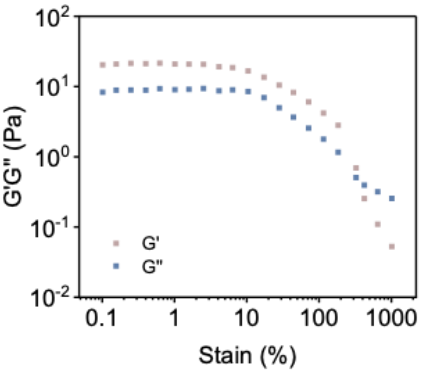

Figure S13. Strain-sweep of c2Y@Au hydrogels at a constant frequency of 10 rad s^{-1} and 37 °C.

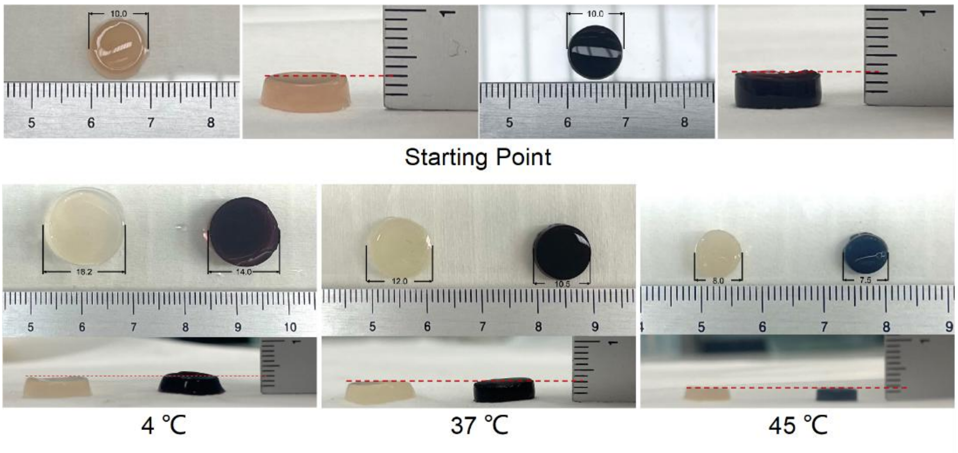

Figure S14. Photos of the swelling and deswelling properties of c2Y (white) and c2Y@Au (black) hydrogels in DI water.

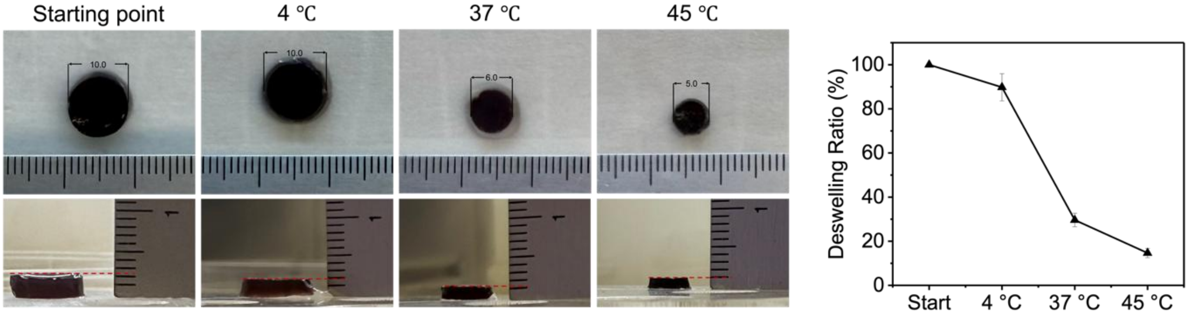

Figure S15. Photos of the deswelling properties of c2Y@Au hydrogels in PBS.

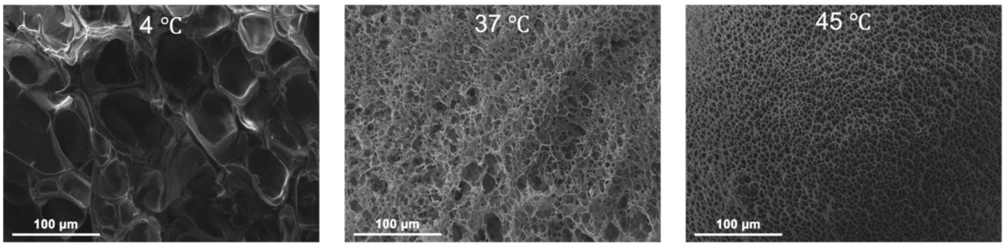

Figure S16. SEM images of c2Y@Au hydrogel cross-sections at 4, 37, and 45 ℃.

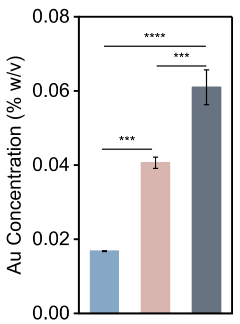

Figure S17. The Au concentrations of c2Y@Au hydrogels from 3 different groups for in vitro photothermal conversion studies. Samples were from 3 different groups prepared for in vitro studies. The comparison among groups is performed using One-way ANOVA (***p < 0.001, ****p < 0.0001).

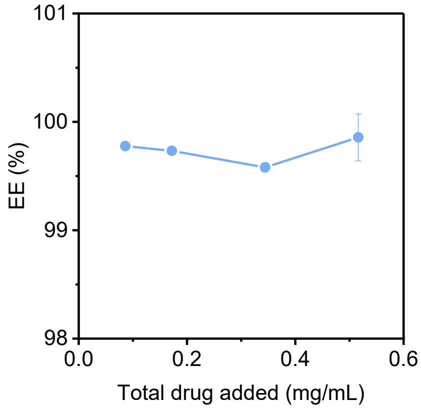

Figure S18. Encapsulation efficiency (EE) of c2Y@Au hydrogels with different DOX loadings.

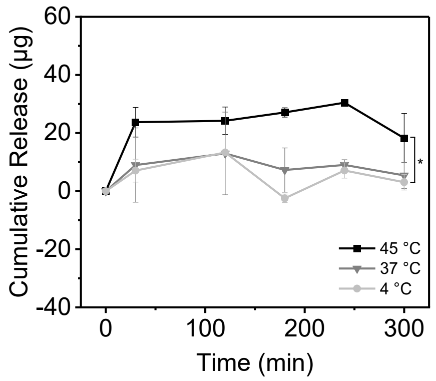

Figure S19. Temperature-dependent drug release profiles of IgG from c2Y@Au hydrogels in water. The comparison among groups was performed using one-way ANOVA (*p < 0.05).

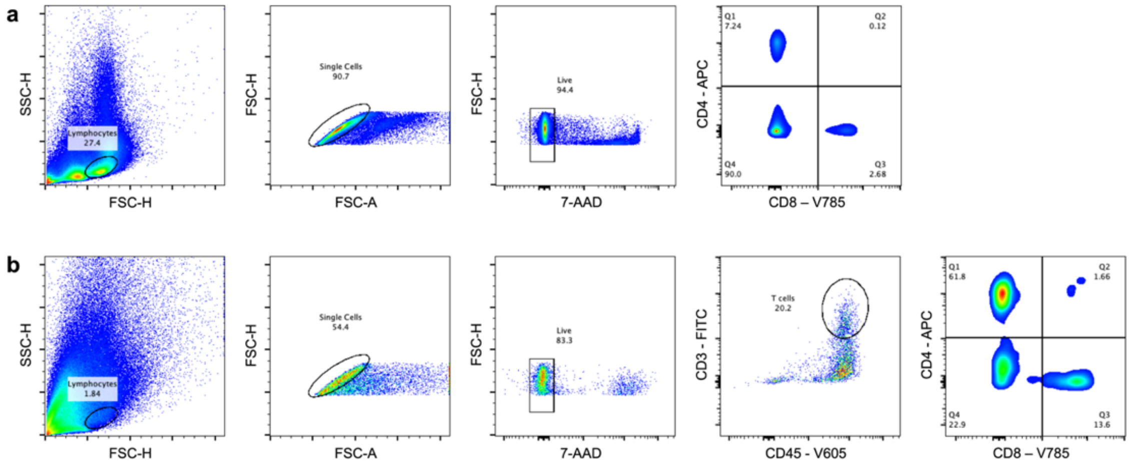

Figure S20. Gating strategies for cell sorting. a) Gating strategy used to sort CD4$^+$ T cells and CD8$^+$ T cells from spleens of 4T1 tumor bearing BALB/C mice. b) Gating strategy used to sort CD3$^+$ (CD45$^+$CD3$^+$) T cells, CD4$^+$ (CD45$^+$CD3$^+$CD4$^+$) T cells, and CD8$^+$ (CD45$^+$CD3$^+$CD8$^+$) T cells from 4T1 tumors.

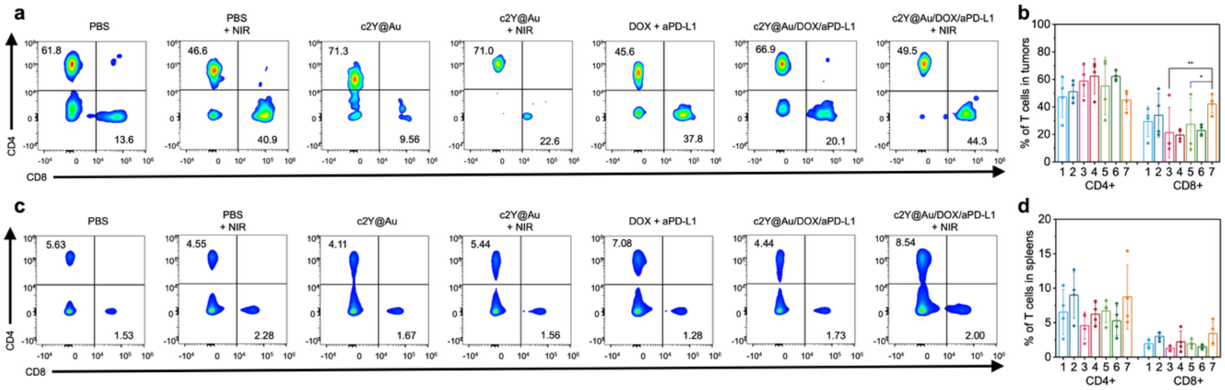

Figure S21. Treatment efficacy of the cSELP-based SPTCI strategy on the single 4T1 tumor model. a, b) Intratumor infiltration of CD4$^+$ and CD8$^+$ T cells (gated on CD3$^+$ T cells). Data represents mean ± sd (n = 4 biologically independent samples). c, d) Percentage of CD4$^+$ and CD8$^+$ T cells in the spleens (gated on live cells). Data represents mean ± sd (n = 4 biologically independent samples). The comparison among groups is performed using One-way ANOVA (*$p < 0.05$, **$p < 0.01$).

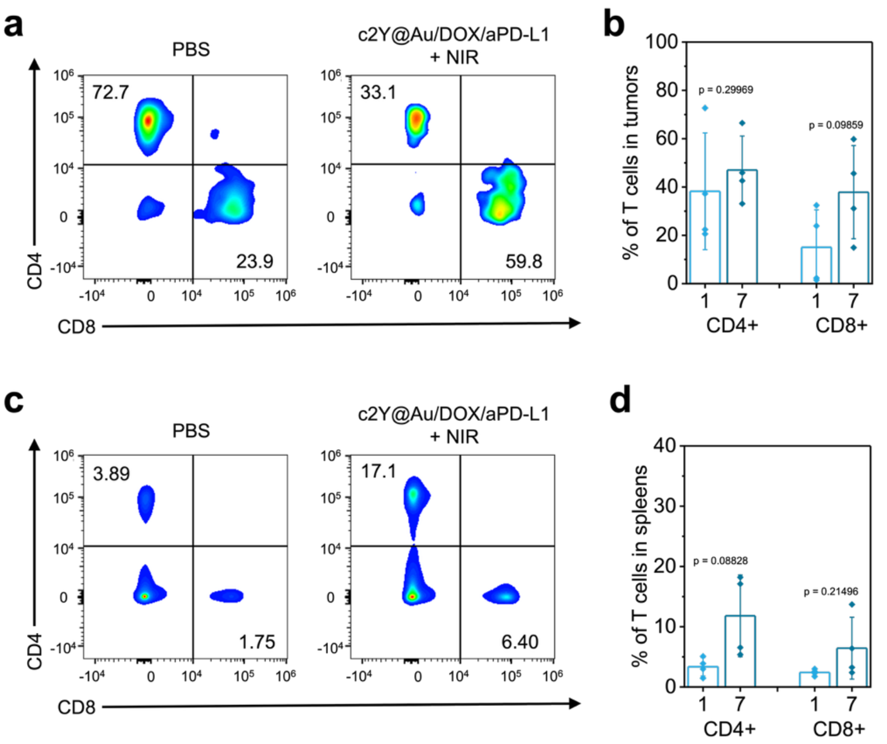

Figure S22. The abscopal effects on tumors using the cSELP-based SPTCI strategy. a, b) Intratumor infiltration of $CD4^+$ and $CD8^+$ T cells (gated on $CD3^+$ T cells). c, d) Percentage of $CD4^+$ and $CD8^+$ T cells in the spleens (gated on live cells). Data represent mean ± sd (n = 4 biologically independent samples). The comparison of each group with the control group is performed using t-test.

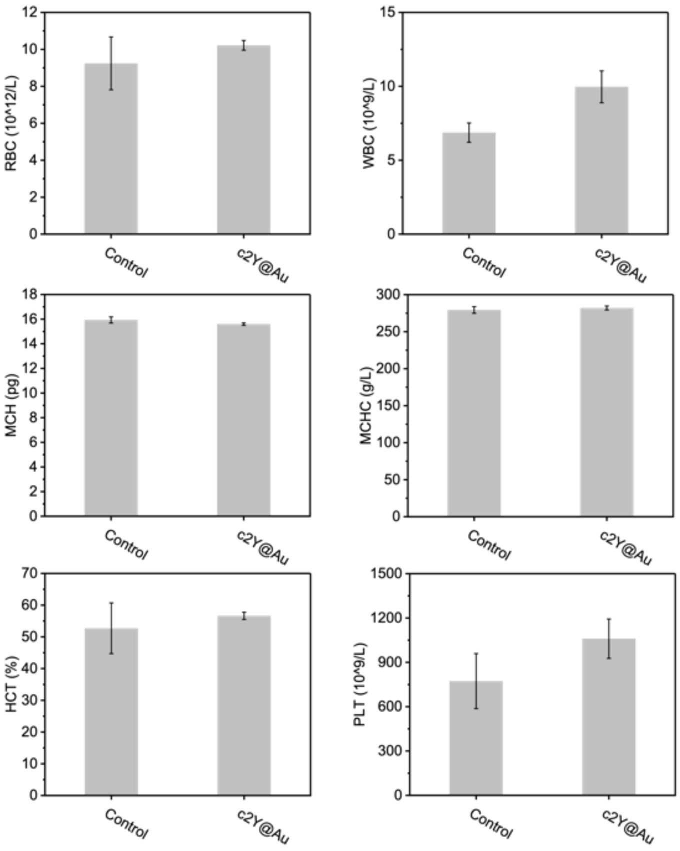

Figure S23. The complete blood count of healthy mice (control group) and healthy mice with subcutaneous injection of c2Y@Au hydrogels (c2Y@Au group). Data represent mean ± sd (n = 3 biologically independent samples). The comparison of each group with the control group is performed using t-test. No significant difference was observed. Abbreviations: red blood cell count, RBC; white blood cell count, WBC; hemoglobin, HGB; mean corpuscular hemoglobin, MCH; mean corpuscular hemoglobin concentration, MCHC; mean corpuscular volume, MCV; platelet, PLT; hematocrit, HCT.

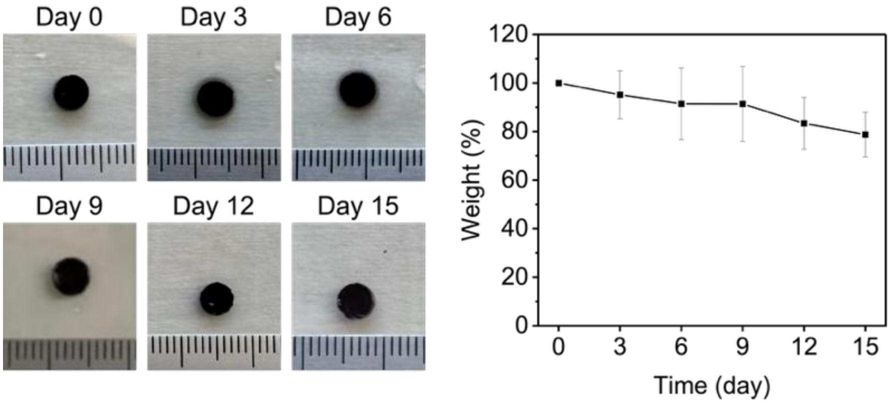

Figure S24. In vitro c2Y@Au hydrogels degradation in PBS at 37 ℃.

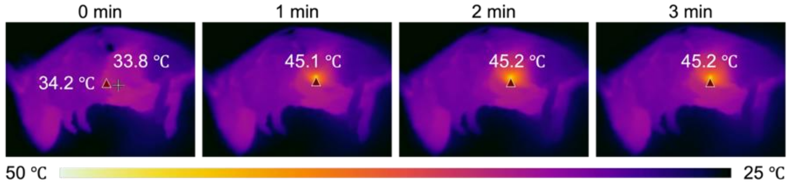

Figure S25. Infrared thermal images of tumor-bearing mice in c2Y@Au/DOX/aPD-L1 + NIR group on day 8, recorded at 0, 1, 2, and 3 min after laser irradiation. Red arrows indicate the highest temperature in the image, while white crosses mark the tumor site temperature.

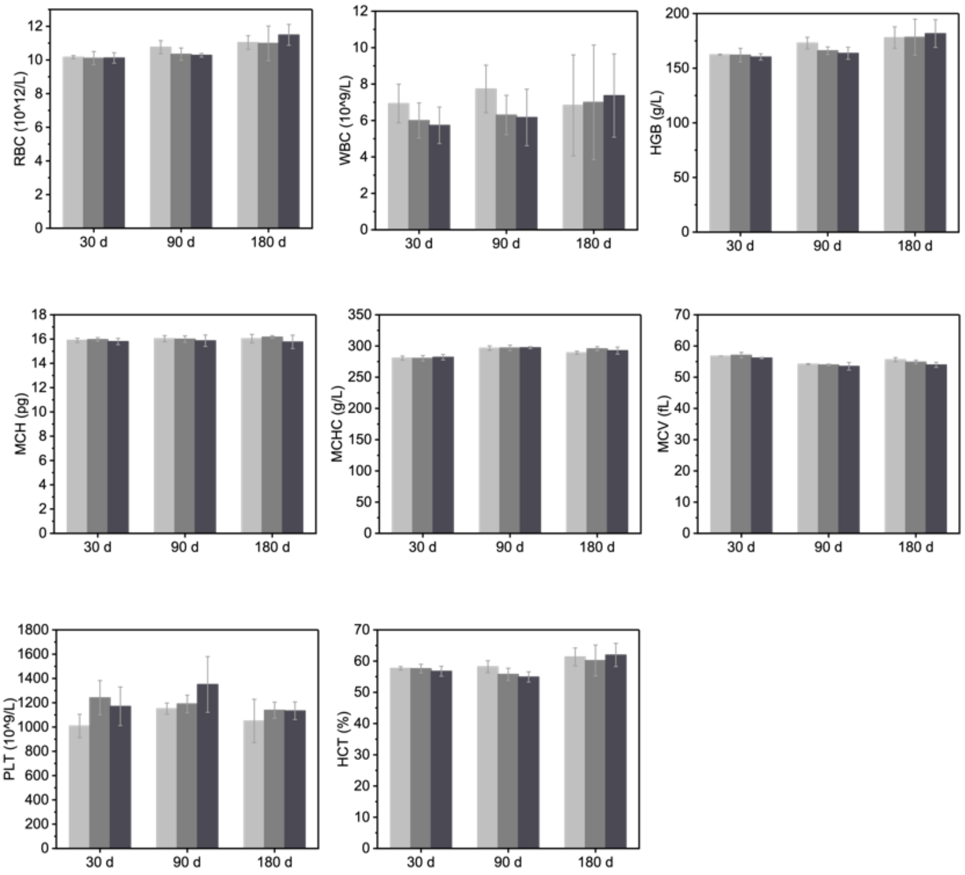

Figure S26. The complete blood count of healthy mice (control group), healthy mice subcutaneously injected with PEG@Au solutions (PEG@Au group), and healthy mice subcutaneously injected with c2Y@Au hydrogels (c2Y@Au group). Samples were collected at 30 days, 90 days, and 180 days after the injection. Data represent mean ± sd (n = 3 biologically independent samples). The comparison among three groups at each time point is performed using one-way ANOVA. No significant difference was observed. Abbreviations: red blood cell count, RBC; white blood cell count, WBC; hemoglobin, HGB; mean corpuscular hemoglobin, MCH; mean corpuscular hemoglobin concentration, MCHC; mean corpuscular volume, MCV; platelet, PLT; hematocrit, HCT.

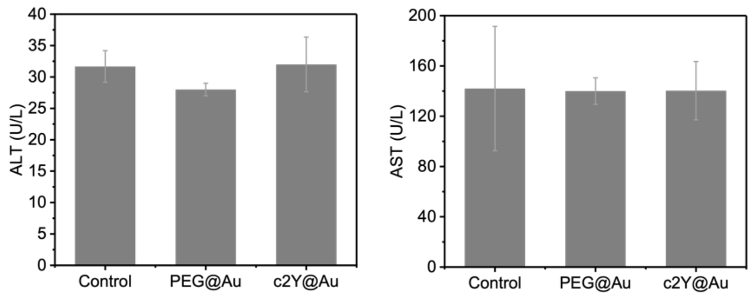

Figure S27. The mice plasma biochemical analysis of healthy mice (control group), healthy mice subcutaneously injected with PEG@Au solutions (PEG@Au group), and healthy mice subcutaneously injected with c2Y@Au hydrogels (c2Y@Au group). Samples were collected 180 days after the injection. Data represent mean ± sd (n = 3 biologically independent samples). The comparison among three groups is performed using one-way ANOVA. No significant difference was observed. Abbreviations: aspartate aminotransferase, AST; alanine aminotransferase, ALT.
